# Locally adapted gut microbiomes mediate host stress tolerance

**DOI:** 10.1038/s41396-021-00940-y

**Published:** 2021-03-03

**Authors:** Shira Houwenhuyse, Robby Stoks, Shinjini Mukherjee, Ellen Decaestecker

**Affiliations:** 1 Laboratory of Aquatic Biology, Department of Biology, University of Leuven—Campus Kulak, E. Sabbelaan 53, B-8500 Kortrijk, Belgium; 2 Evolutionary Stress Ecology and Ecotoxicology, University of Leuven, Charles Deberiotstraat 32, 3000 Leuven, Belgium; 3 Laboratory of Aquatic Ecology, Evolution and Conservation, University of Leuven, Charles Deberiotstraat 32, 3000 Leuven, Belgium

**Keywords:** Microbial ecology, Microbial ecology, Freshwater ecology, Microbiome

## Abstract

While evidence for the role of the microbiome in shaping host stress tolerance is becoming well-established, to what extent this depends on the interaction between the host and its local microbiome is less clear. Therefore, we investigated whether locally adapted gut microbiomes affect host stress tolerance. In the water flea *Daphnia magna*, we studied if the host performs better when receiving a microbiome from their source region than from another region when facing a stressful condition, more in particular exposure to the toxic cyanobacteria *Microcystis aeruginosa*. Therefore, a reciprocal transplant experiment was performed in which recipient, germ-free *D. magna*, isolated from different ponds, received a donor microbiome from sympatric or allopatric *D. magna* that were pre-exposed to toxic cyanobacteria or not. We tested for effects on host life history traits and gut microbiome composition. Our data indicate that *Daphnia* interact with particular microbial strains mediating local adaptation in host stress tolerance. Most recipient *D. magna* individuals performed better when inoculated with sympatric than with allopatric microbiomes. This effect was most pronounced when the donors were pre-exposed to the toxic cyanobacteria, but this effect was also pond and genotype dependent. We discuss how this host fitness benefit is associated with microbiome diversity patterns.

## Introduction

Many organisms face different environmental conditions across their species’ range and thereby evolved local adaptation with organisms being relatively fit in their local conditions and relatively unfit in others [1, 2]. Local adaptation is often studied in macro-organisms across large-scale environmental gradients [3–7]. Nevertheless, it can also occur at much smaller geographical scales and in micro-organisms, as shown for parasites and microbiomes [8–11]. While there is ample evidence for local adaptation among natural populations, it is not always detected [8–10]. One reason may be that local adaptation is widely considered to be the result of fitness trade‐offs across environments [11, 12], which may be more likely to be detected under stressful conditions that limit energy stores [13–16]. Local adaptation may, however, also truly be absent because of out-of-phase dynamics in coevolutionary processes, a weak response to selection, and gene flow between host populations [9, 17–19].

Insights in the genomic targets and the molecular mechanisms underpinning local adaptation are starting to accumulate thanks to the increased affordability of high-throughput sequencing [20–22]. However, several challenges, such as the identification of the true targets of local adaptation still remain [23]. Recently, the gut microbiome of different organisms has emerged as a key determinant of many aspects of organismal biology, capable of shaping developmental, physiological and reproductive phenotypes [24–29]. The composition of gut microbiomes differs spatially in function of the regional environmental conditions [30–34], and there is some evidence that different host genotypes differ in their gut microbiome [35, 36]. It is therefore likely that when hosts show local genetic adaptation, this may be misleading as their gut microbiome may, at least partly, be contributing to this pattern [37]. There is recent evidence that the gut microbiome plays a role in local adaptation to environments differing in, for example, pollution [38], aridity [39] and salinity [40, 41]. These studies, however, mainly focused on how the microbiome differed between locations, but did not explicitly investigate the host fitness effects caused by carrying a local microbiome.

**Fig. 1:**
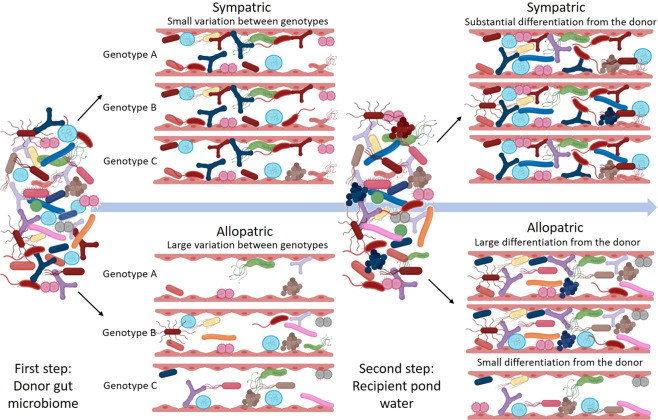
Hypothetical two-step establishment of bacterial strains in recipient Daphnia genotypes from the sympatric vs. allopatric microbial donor inocula. We tested and found support in our data for a higher convergence in the microbial community of the different *D. magna* genotypes in the sympatric than in the allopatric treatment, and this effect was only present when the donors were pre-exposed to *M. aeruginosa*. If *D. magna* individuals recruited a specific, beneficial microbiome (based on Macke et al.) [42, 43], the recipient gut should be occupied by adapted, beneficial bacteria of the donor inoculum. We hypothesize that this effect should be stronger in the sympatric than in the allopatric treatment and especially in the toxic *Microcystis* pre-exposure in the donors, because then selection pressure is assumed to be highest, resulting in a more convergent microbial community over the different genotypes in the sympatric than in the allopatric treatment. If *D. magna* recipient individuals received an allopatric microbiome, donor bacteria should enter and establish randomly in the recipient guts. This will result in variable microbial communities that (1) can be the same as the donors (so low dissimilarity), but (2) can also be totally different (obtained from the environmental pond water pool). Hence, variation in the microbial communities between the different host genotypes in the allopatric treatments is expected to be much higher than in the sympatric treatments.

In this study, we tested through reciprocal transplants, whether having a sympatric vs. an allopatric gut microbiome gives the host an advantage in stressful environments. The underlying hypothesis is that hosts profit from having a locally adapted microbiome, which increases their fitness under stressful conditions. This was hypothesized to be associated with two different scenarios in terms of gut microbiome diversity and establishment. Based on Macke et al. [42, 43], *Daphnia magna* selects beneficial bacteria from the environment. We expect stronger host-mediated selection for beneficial bacterial strains in sympatric than in allopatric microbiomes. If so, the gut will be occupied by locally selected bacteria leaving less opportunity for other bacteria to establish, which may result in more convergent microbiomes over the host genotypes (see Fig. 1) [44]. Alternatively, hosts with a sympatric microbiome can be expected to have a larger bacterial spectrum with beneficial gene functions [45], hence showing a higher bacterial diversity than with an allopatric microbiome.

To unravel whether a local microbiome could play a role in increased tolerance, *D. magna* genotypes from different ponds out of two separate regions were exposed to toxic cyanobacteria in a reciprocal transplant experiment in which germ-free *D. magna* were inoculated with a local or foreign gut microbiome. The zooplankter *D. magna* is an ecological and evolutionary model organism for which local adaptation has been shown in response to stressors, among which cyanobacteria [46–48]. A commonly occurring cyanobacterium genus is *Microcystis*, which is poor food, interferes with *Daphnia* filtering activity, and produces a wide range of deleterious cyanotoxins [49–51]. Because *Microcystis* exposure is stressful for *Daphnia*, exposing *Daphnia* to it makes it likely to detect trade-offs and local adaptation in the host. Because gut microbiomes pre-exposed to *Microcystis* have been shown to provide a higher tolerance to *Microcystis* for the *D. magna* host [42, 43], we tested whether local adaptation in the recipients was dependent on pre-exposure of the donors to toxic *Microcystis aeruginosa*. We addressed following questions: (1) Is fitness higher in *D. magna* receiving a sympatric than an allopatric microbiome and does this depend on the donor diet? (2) Do *D. magna* have a lower or higher microbial diversity when they receive a sympatric vs. an allopatric microbiome? and (3) how is this related to their fitness and genotype?

## Materials and methods

### Sampling of the *Daphnia magna* genotypes and pond water

Five *D. magna* genotypes from each of two regions in Belgium separated by circa 100 km (Kortrijk and Leuven) were collected (Table SI1). In Kortrijk, we obtained *D. magna* genotypes from the Blauwe Hoeve (K_BH), Kennedypark (K_KP) and Morinnestraat (K_MS), by sampling one individual from the active population in each pond. In addition, two genotypes were obtained from a recent (top layer) resting eggs in Zwevegem (K_ZWE1 and K_ZWE2) as at the time of sampling no active population of *D. magna* was present. In Leuven, all genotypes were obtained from recent (T = top layer sediments) resting eggs of two ponds: Heverlee (L_OM2) and Oud-Heverlee (L_T2, L_T3, L_T7 and L_T8; coding is based on Cousyn et al. [52]). After being kept as stock cultures, they were raised as three independent clonal (maternal) lines (i.e., genetically identical *Daphnia* individuals raised independently from each other to control for maternal effects). The ponds from Kortrijk out of which the genotypes were sampled did not have a *Microcystis* bloom at sampling (S. Houwenhuyse, personal observation), but in some ponds of Leuven (i.e., L_OM2) *Microcystis* blooms have been observed (E. Decaestecker, personal observation). It could be possible for the Leuven genotypes to be locally adapted to cyanobacteria. However, *Daphnia* adaptation to *Microcystis* strains is assumed to be locally structured [53], and we here test the role of a local vs. a foreign microbiome towards a ‘neutral’ toxic cyanobacterial strain. Hence, irrespective of the absence/presence of cyanobacteria in the ponds, no adaptation to the here tested toxic cyanobacterial strain was expected in *Daphnia*. Maternal lines of the *D. magna* genotypes were cultured in filtered tap water at a temperature of 19 ± 1 °C and under a 16:8 h light:dark cycle in 2 L glass jars (at a density of 30 individuals/L). They were fed three times per week with saturating amounts of the green algae *Chlorella vulgaris*. The medium of these cultures was refreshed once per week. Three months before the start of the experiment 10 L pond water was collected from each pond and stored at 4 °C to maintain the bacterioplankton community. At the time of pond water sampling, there was no visible presence of cyanobacteria in any pond. After sieving the pond water over a 100 µm sieve, two mixtures (=filtered local pond water) were made, one from the Kortrijk ponds, and one mixture from the Leuven ponds to be inoculated in the experimental jars during the transplant experiment.

### Cultivation of green algae and cyanobacteria

*D. magna* individuals were fed with the unicellular green algae *Chlorella vulgaris* (which is good-quality food for *D. magna* [54]) or a combination of *C. vulgaris* and *M. aeruginosa* (which is a toxic cyanobacterial strain for *D. magna* [42]). We used the *M. aeruginosa* strain PCC 7806, isolated from the Braakman reservoir in the Netherlands (51°19′22″ N, 3°44′16″ E) and part of the Culture Collections at Institute Pasteur (Paris, France). *C. vulgaris* and *M. aeruginosa* were grown in Wright’s Cryptophyte (WC) medium [55] and modified WC medium (without Tris), respectively. The algae were cultured under sterile conditions in a climate chamber at 22 ± 1 °C with a light:dark cycle of 16:8 h in 2 L glass bottles, with constant stirring and aeration. Filters (0.22 µm) were placed at the input and output of the aeration system to avoid any bacterial contamination. The axenity of the algal cultures was checked on Lysogeny Broth medium [56] agar plates. Ash-free dry weight of the cultures was determined following Moheimani et al. [57].

### Gut microbiome transplant experiment

**Fig. 2:**
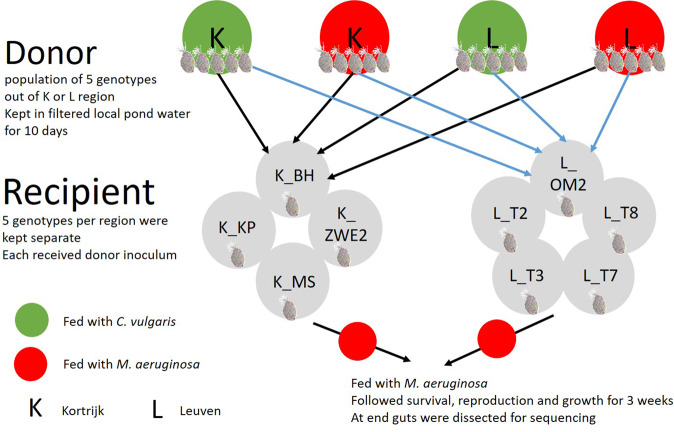
Experimental design. Five *D. magna* genotypes from each of two regions (Kortrijk and Leuven, respectively K and L) were collected together with their pond water. In the donor phase the five genotypes were pooled per region and kept in the lab in the pond water from their region. Half of the donor populations were fed with the toxic laboratory strain *Microcystis aeruginosa*, the other half was fed with a control diet *Chlorella vulgaris*. After 10 days, guts from these donor populations were dissected and used as in inoculum. In the recipient phase, the five (and four, one of the K genotypes—K_ZWE1—did not hatch after sterilization) genotypes per region were made germ-free and received a sympatric vs. an allopatric donor microbiome that was pre-exposed to either *C. vulgaris* or *M. aeruginosa*. These recipient *D. magna* were then fed with *M. aeruginosa*. Survival, fecundity and growth were followed for 3 weeks. After 3 weeks the guts were dissected for amplicon sequencing. In total eight genotypes were characterized on their microbiome profile: four K and four L genotypes (L_T2 did not survive until day 21).

To determine if *D. magna* shows a better performance upon exposure to toxic cyanobacteria in the presence of a sympatric vs. an allopatric gut microbiome, a reciprocal gut microbiome transplant experiment was conducted (Fig. 2). We first exposed donor individuals to *M. aeruginosa* to test whether pre-exposure of the microbiome results in a higher stress tolerance in the recipients. Donor genotypes were pooled per region (five genotypes per region), per algal treatment (pre-exposed to a mixture of 60% toxic *M. aeruginosa* and 40% *C. vulgaris*, or to 100% *C. vulgaris*) and per replicate and were kept at a density of 50 (i.e., ten per genotype) individuals in 2 L experimental jars with filtered local pond water. Donor diets had a final carbon concentration of 1 mg C/L and were given to the *D. magna* three times per week. The pooled donor *D. magna* genotypes stayed in these conditions for 10 days to adjust the gut microbiome when exposed to *M. aeruginosa* [42]. Afterwards, 25 *D. magna* individuals per donor region × diet combination were randomly isolated and kept in sterile-filtered tap water for 24 h to remove food particles from the gut, as well as environmental bacteria on the carapace and filter apparatus. Then, guts were extracted with dissection needles under a stereo microscope and placed in an Eppendorf tube containing 1 mL sterile milliQ water. These dissected guts were then stored at 4 °C for 7 days. Each donor condition was set up in triplicate, which resulted in 12 pooled donor inocula (two donor regions × two donor diets × three replicates). After 7 days, the stored donor inocula were crushed and given to sterile *D. magna* juveniles. In the recipient phase, each genotype received a pooled and crushed gut microbiome of one of the four donor treatment combinations. This resulted in a fully crossed design: donor region (two levels) × donor diet (two levels) × *D. magna* genotype (nested within two recipient regions, with four genotypes for Kortrijk as one genotype did not hatch after sterilization and five genotypes for Leuven) with three replicates per genotype (one per independent maternal line). In sympatric combinations *D. magna* received a local donor inoculum, in allopatric combinations they received a foreign donor inoculum. Sterile *D. magna* juveniles were obtained following the protocols of Callens et al. [58, 59]. Therefore, per maternal line of each genotype, 30 parthenogenetic eggs with external membranes were collected in a six-well plate with 15 eggs in 5 mL filtered tap water per well. Afterwards, they were placed in a laminar flow hood to disinfect the eggs by submersing them in 5 mL of a 0.1% glutaraldehyde solution and gently agitating them for 10 min. Then, the eggs were transferred to another well, containing 5 mL sterile filtered tap water to remove glutaraldehyde residues. The eggs stayed in this rinsing step for 10 min after which the rinsing step was repeated. Afterwards, the eggs were transferred in groups of 15–5 mL sterile filtered tap water. The six-well plate was sealed with parafilm and placed in an incubator. Eggs were allowed to hatch for 72 h at a temperature of 20 ± 0.5 °C and a 16:8 h light:dark cycle. The genotype K_ZWE1 did not hatch after sterilization and therefore excluded from the recipient phase of the experiment, resulting in a total of nine recipient genotypes. Per maternal female line, eight germ-free juveniles were placed per two in 50 mL falcon tubes filled with 45 mL sterile filtered tap water. Each of those pairs received one of the four different pooled donor inocula. The recipient *D. magna* were incubated with the pooled donor inocula for 48 h, after which *D. magna* were individually transferred to new 50 mL falcon tubes filled with filtered local pond water. In these 48 h, the recipient *D. magna* will have taken up a substantial part of the bacteria from the pooled donor inoculum. After the transfer to (unsterile) filtered local pond water (filtered over a 100 µm sieve), all recipient *D. magna* were subjected to a cyanobacterial diet (i.e., 60% toxic *M. aeruginosa* and 40% *C. vulgaris*), which was administered every 48 h to test if there was an effect from the donor gut microbiome on *Daphnia* tolerance to toxic cyanobacteria in terms of host fitness, survival and reproduction, monitored every 48 h during 21 days. Body size was measured at days 3, 6, 10 and 20. After 21 days, the guts from the surviving *D. magna* were dissected for amplicon sequencing. The *D. magna* were placed in sterile-filtered tap water for 24 h to remove food particles from the gut, as well as environmental bacteria on the carapace and filter apparatus. After these 24 h, they were dissected under a stereo microscope using dissection needles and collected in 10 µL sterile milliQ water, after which they were stored in −20 °C.

### Library preparation and sequencing

To characterize the gut microbial communities from the donor sets and from the recipient *D. magna* at the end of the 21-day monitoring period, DNA was extracted using the PowerSoil DNA isolation kit (Qiagen). DNA was dissolved in 20 µL milliQ water. The total DNA yield was determined using a Qubit dsDNA HS assay (Invitrogen) on 1 µL of sample. A nested PCR was applied to increase specificity and amplicon yield. The full-length 16S rRNA gene was first amplified with EUB8F and 1492R primers on 10 ng of template using a high-fidelity SuperFi polymerase (Life Technologies) for 30 cycles: 98 °C—10 s; 50 °C—45 s; 72 °C—30 s. PCR products were subsequently purified using the QIAquick PCR purification kit. To obtain dual-index amplicons of the V4 region, a second amplification was performed on 5 µL (=20–50 ng) of PCR product using 515F and 806R primers for 30 cycles: 98 °C—10 s; 50 °C—5 s; 72 °C—30 s. Both primers contained an Illumina adapter and an 8-nucleotide (nt) barcode at the 5′-end. For each sample, PCRs were performed in triplicate, pooled and gel-purified using the QIAquick gel extraction kit. An equimolar library was prepared by normalizing amplicon concentrations with a SequalPrep Normalization Plate (Applied Biosystems) and subsequent pooling. Amplicons were sequenced using a v2 PE500 kit with custom primers on the Illumina Miseq, producing two ×250-nt paired-end reads. This way, 96 recipient samples were generated representing eight genotypes (one of the nine recipient genotypes, L_T2, did not have any surviving individuals after 21 days) × two donor regions × two donor diets × three replicates, and the 12 donor samples. The number of sequenced *D. magna* individuals per treatment is represented in the Table SI2.

DNA sequences were processed following Callahan et al. [60]. Sequences were trimmed (the first ten nucleotides and from position 190 onwards) and filtered (maximum of two expected errors per read) on paired ends jointly. Sequence variants were inferred using the high-resolution DADA2 [61] method, which relies on a parameterized model of substitution errors to distinguish sequencing errors from real biological variation. Chimeras were subsequently removed from the dataset. After filtering, a total of 907,846 reads were obtained with on average 24,536.38 reads per sample (minimum = 23 reads and maximum = 53,480 reads), with most samples having more than 10,000 reads (only four exceptions with 23, 9080, 8478 and 3840 reads). Taxonomy was assigned with a naïve Bayesian classifier using the Silva v132 training set. OTUs with no taxonomic assignment at the phylum level or which were assigned as “chloroplast” or “cyanobacteria” were removed from the dataset. OTUs for which the mean relative abundance was below 10^−5^ were removed from the analysis. To visualize the bacterial families that differed between the treatments, OTUs were grouped at the family level, and families representing <1% of the reads were discarded (this was not done for the analyses). To test for differences in α- and β- diversity, all samples were rarefied to a depth of 1000 reads. One gut sample (KP, with a sympatric donor microbiome pre-exposed to *M. aeruginosa*) had a lower number of reads, and was removed from the analysis.

### Statistical analyses

Statistical analyses of the life history traits were performed in R 4.0.0 (R studio version 1.1.463). We used the Akaike information criterion (AIC) to select the best subset of variables to represent the best model (Table SI3). We evaluated general linear models (GLM with Gaussian or normal distribution, as the data was normally distributed) and linear mixed-effects models (LMER). We first compared models with the same fixed effects, but different random effect (i.e., (1|Region:Pond:Genotype) and (1|Region:Pond)). Secondly, we tested the significance of the fixed factors in the model with the best random effects factor. Type II ANOVA tables for fixed-effect terms with Satterhwaite and Kenward–Roger methods for dominator degrees of freedom for *F*-tests and *p* values were created (Anova function of the car package [62]). In the final model, we included donor diet (toxic cyanobacteria absent or present), donor microbiome type (allopatric or sympatric microbiome), and *D. magna* genotype (=clone) as fixed factors, with genotype nested within pond (see Table SI1) and pond nested within region as random factor. We also included all possible interactions. Survival was analyzed in two different ways, first using a log-rank or Mantel-Haenszel test (from the OIsurv, survival and survminer packages in R [63]) on a random selection of one genotype per pond (fully randomized over the genotypes, and thus correcting for dependency between genotypes within the L2 pond) and on a total of nine recipient genotypes. The survival times of individuals that were still alive at the end of the 21-day experiment were coded as right-censored. The second way the survival was analyzed was on the percentage *D. magna* individuals that survived until the end of the experiment. This was done with a general linear mixed-effects model (from the lme4 package in R [64]), controlling for unbalanced design with a restricted maximum likelihood estimation. Total fecundity and body size were analyzed on a total of eight recipient genotypes. Total fecundity was analyzed for these *D. magna* individuals that survived until day 21 with a linear mixed-effects model, controlling for unbalanced design with a restricted maximum likelihood estimation. Body size was analyzed with a linear mixed-effects model with the four subsequent measurements (days 3, 6, 10 and 20) as repeated measures and also controlled for unbalanced design with a restricted maximum likelihood estimation. To specifically analyze relationships in the response patterns to *M. aeruginosa* pre-exposure between traits, we calculated delta values as the clonal average trait value when pre-exposed to *M. aeruginosa*—the clonal average trait value when not pre-exposed. We did so for survival, total fecundity and body size (days 3, 6, 10 and 20). We then tested with a Pearson correlation test, with bonferroni correction for multiple testing (using the ggpubr package in R [65]), for pairwise relationships between delta values of different traits (e.g., between survival and body size).

Measures for α-diversity of the gut microbial communities within the different treatments, OTU richness and the Shannon entropy (taking into account both OTU richness and the relative abundance of OTUs) were calculated using the vegan package in R [66]. Shannon entropy was calculated as the exponential function of the Shannon entropy, this represents the true diversity of the bacterial community in the sample [67, 68]. We used the AIC criterion to select the best subset of variables to represent the best model (Table SI3). We evaluated GLM (with Gaussian or normal distribution, as the data was normally distributed) and LMER. We first compared models with the same fixed effects, but different random effect (i.e., (1|Region:Pond:Genotype) and (1|Region:Pond)). Secondly, we tested the significance of the fixed factors in the model with the best random effects factor. Type II ANOVA tables for fixed-effect terms with Satterhwaite and Kenward-Roger methods for dominator degrees of freedom for *F*-tests and *p* values were created (Anova function of the car package [62]). In the final model, the effects of donor diet, microbiome type, genotype, and all possible interactions, were assessed through a linear mixed-effects model, controlling for unbalanced design with a restricted maximum likelihood estimation with as random factor genotype nested in pond and pond nested within recipient region on a total of eight recipient genotypes. To investigate differences in community composition (β-diversity) between the different microbial communities, Bray–Curtis dissimilarity indices were calculated and plotted using principal coordinates analysis with the phyloseq package in R [69]. The effects of donor diet, microbiome type, genotype, and all possible interactions, with random factor genotype nested in pond and pond nested within recipient region on β-diversity were assessed through a permutation MANOVA, using the Adonis2 function in the vegan package in R [70] on the total dataset (i.e., donors and recipients), and on the donors and the recipients separately. To identify bacterial families that differed between donors and recipients, between diets and between microbiome types, OTUs were grouped at the family level, and families representing <1% of the reads were discarded. Differential abundance analyses were then performed with the Bioconducter package DESeq2 [71]. A Unionplot was created using the wilkox/unionplot function from GitHub, to show the OTUs that are unique and shared between donor, allopatric and sympatric treatments. In addition, the Bray Curtis dissimilarity matrix was extracted to perform an ANOVA on this matrix, to determine the difference in distance between donor and recipients across the treatments. Based on AIC the best model was a general linear model with diet, microbiome type and genotype as fixed factors.

## Results

Given the complexity of the results, we only focus on significant effects in the text below. For full results, see Tables SI 1–12.

### D. magna life-history traits

**Fig. 3:**
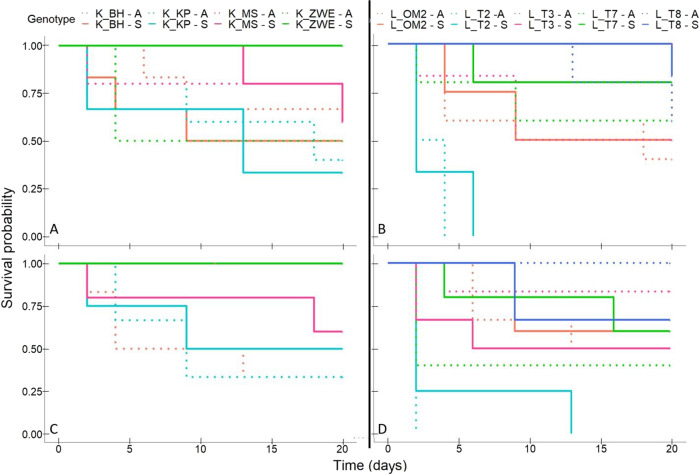
*D. magna* survival in function of the donor diet and microbiome type. Allopatric (A-extension in the figure legend, dotted line) or Sympatric (S-extension in the figure legend, full line) microbiome: four Kortrijk (K) *D. magna* genotypes that received a microbiome **A** pre-exposed to a toxic *M. aeruginosa* strain or **C** not pre-exposed to *M. aeruginosa*; five Leuven (L) *D. magna* genotypes that received a microbiome **B** pre-exposed to a toxic *M. aeruginosa* strain or **D** not pre-exposed to *M. aeruginosa*.

**Fig. 4:**
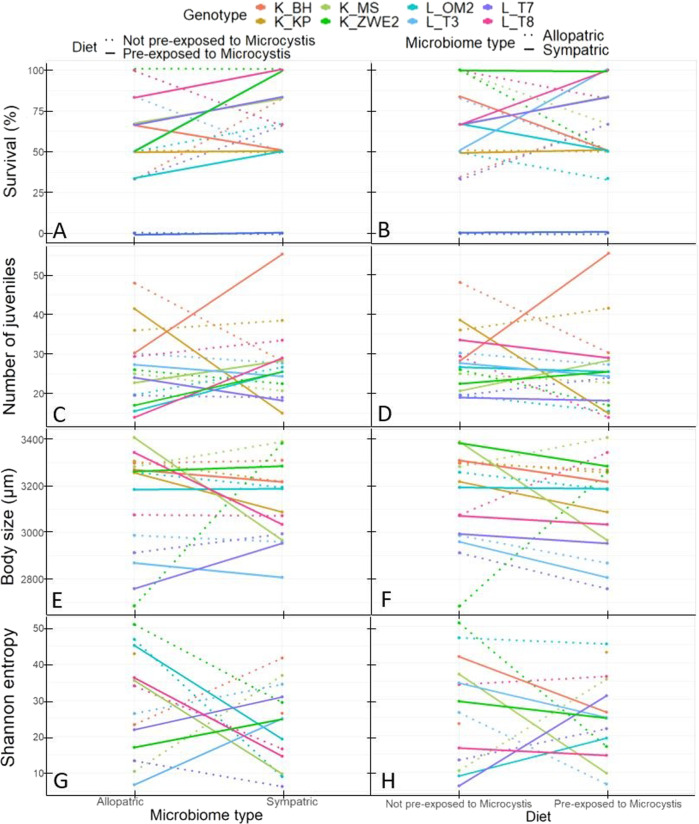
Interaction plots representing the three-way interaction for survival, reproduction, body size and Shanonn entropy. Interaction plots representing: percentage survived *D. magna* at day 20 (**A**, **B**), total fecundity (**C**, **D**), body size at day 20 (**E**, **F**) and exponential value of the Shannon entropy (**G**, **H**) of the *D. magna* genotypes. Left column shows variable in function of microbiome type (allopatric vs. sympatric) with donor microbiomes pre-exposed to *M. aeruginosa* in full lines and donor microbiomes not pre-exposed to *M. aeruginosa* in dotted lines. Right column shows variable in function of diet (pre-exposed or not to *M. aeruginosa*) with sympatric microbiomes in full lines and allopatric microbiomes in dotted lines.

For survival, the three-way interaction between donor diet (pre-exposed to *M. aeruginosa* or not), donor microbiome type (sympatric vs. allopatric) and recipient pond, was highly significant (*X²* = 57.9, df = 23, *p* < 0.0001; Table SI4). When tested separately per donor diet and using one genotype per pond, there was a significant recipient genotype × donor microbiome type only when the host received a microbiome pre-exposed to the toxic *M. aeruginosa* strain (*X²* = 23.7, df = 11, *p* = 0.01), but not when the microbiome was not pre-exposed to *M. aeruginosa* (*X²* = 16.2, df = 11, *p* = 0.1). When the microbiome was pre-exposed to the toxic *M. aeruginosa* strain, seven out of the nine genotypes (K_MS, K_ZWE2, L_OM2, L_T2, L_T3, L_T7 and L_T8) had a higher survival percentage when they received a sympatric than an allopatric microbiome, for the other two genotypes (K_BH, K_KP) the survival percentage was higher when they received an allopatric than a sympatric microbiome (Fig. 3, Table SI5). This result is confirmed when looking at the interaction plots (Fig. 4A, B), most of the genotypes had a higher survival when they received a sympatric microbiome pre-exposed to *M. aeruginosa*.

For *D. magna* fecundity, there was a significant three-way interaction between donor diet × donor microbiome type × genotype (*F* = 2.6; df = 7, 63; *p* = 0.02; Table SI6 and Fig. SI1). Separate analyses per donor diet showed no significant interaction between microbiome type and genotype for each diet. There was, however, a marginal significant diet x microbiome type (*F* = 3.8; df = 1, 63; *p* = 0.056) reflecting that when *D. magna* individuals received a microbiome that was pre-exposed to the toxic *M. aeruginosa* strain more juveniles were produced when they received a sympatric than an allopatric microbiome (Fig. 4C, D and Fig. SI2). This was not the case when the donor inoculum was not pre-exposed to *M. aeruginosa*, there less juveniles were reproduced when they received a sympatric than an allopatric microbiome. Separate analysis, with a model excluding the fixed factor genotype, showed that the diet x microbiome type interaction was dependent on the genotype, as the interaction between diet and microbiome type was no longer significant in this model (*F* = 2.7; df = 1, 80; *p* = 0.1).

For *D. magna* body size, there was a three-way interaction between donor diet, microbiome type, and genotype (Table SI7). Separate analysis per donor diet showed that when *D. magna* received a microbiome pre-exposed to *M. aeruginosa* there was no significant interaction between microbiome type and genotype (*F* = 1.6; df = 7, 241; *p* = 0.13). However, when the *D. magna* received a microbiome not pre-exposed to *M. aeruginosa* there was a marginally significant interaction between donor microbiome and genotype (*F* = 1.9; df = 7, 219; *p* = 0.066). In addition, there was a significant interaction between donor diet and microbiome type across the repeated size measurements (*F* = 6.5; df = 1, 248; *p* = 0.011). *D. magna* receiving a donor microbiome pre-exposed to *M. aeruginosa* had a smaller body size than when not pre-exposed to *M. aeruginosa*, and this effect was most pronounced when they received a sympatric microbiome (Fis. 4E, F and Fig. SI3). This diet x microbiome type interaction was not dependent on the genotype. A separate analysis, with a model excluding the fixed factor genotype, showed that the diet x microbiome type interaction was still significant (*F* = 6.5; df = 1, 352; *p* = 0.01). There was also a marginally significant interaction between microbiome type and genotype (*F* = 1.9; df = 7, 617; *p* = 0.057, Fig. SI4), and time and genotype (*F* = 2.04; df = 21, 248; *p* = 0.005) for *D. magna* body size, and a significant main effect of genotype (*F* = 5.9; df = 7, 648; *p* < 0.0001) and time (day 3, 6, 10 and 20; *F* = 1114; df = 3, 248; *p* < 0.0001).

**Fig. 5:**
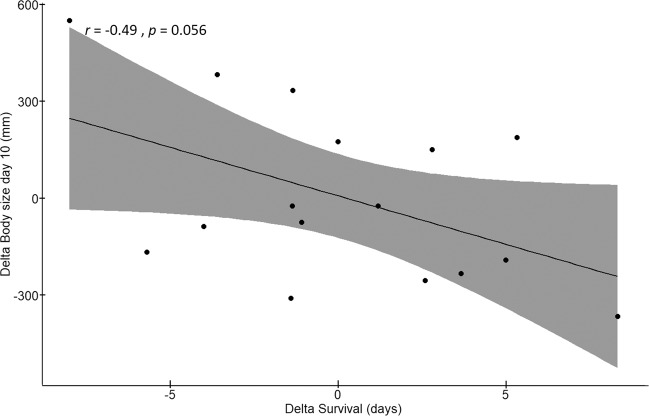
Results of the Pearson correlation test between the delta (average *Microcystis* per genotype − average control per genotype) of survival and body size at day 10. *r* represents the Pearson’s correlation coefficient between survival and body size at day 10. *p* represents the *p* value of the Pearson correlation test.

Analysis of the differences between control and cyanobacterial donor diet revealed a marginally significant negative correlation between the *D. magna* survival and body size (at day 10) of the recipients (Pearson correlation using the average per genotype: *r* = −0.48, *t* = −2.08, df = 14, *p* = 0.055; Table SI8) reflecting that genotypes that showed a more positive response in survival tended to show a more negative response in body size, when they received a microbiome that has been pre-exposed to toxic *M. aeruginosa* (Fig. 5).

### Gut microbiome composition

**Fig. 6:**
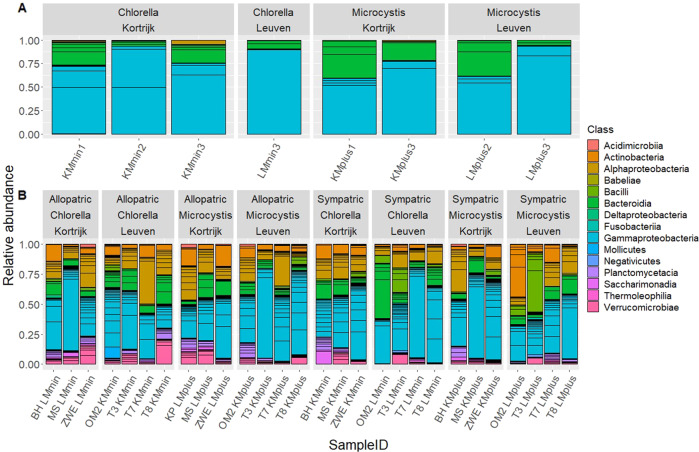
Relative abundance of bacterial classes in de donor and recipient gut microbiomes. Relative abundance of bacterial classes in the gut microbiomes of the four donor sets (**A**) and the recipient genotypes (**B**), grouped by location of origin, donor diet and donor microbiome type. Colors indicate different bacterial classes. OTUs with an occurrence lower than 1% are not represented. K Kortrijk, L Leuven, Mplus donor microbiome pre-exposed to *M. aeruginosa*, Mmin donor microbiome not pre-exposed to *M. aeruginosa*.

The donor microbiomes consisted mainly of Gammaproteobacteria and Bacteroida (Flavobacteriia). The recipient gut microbiomes also harbored Gammaproteobacteria and Bacteroida, but were more diverse than the donor microbiomes (Shannon entropy: *F* = 201; df = 1, 30; *p* < 0.0001; Fig. SI5) and contained also bacteria from following classes: Actinobacteria, Alphaproteobacteria, Bacilli, Planctomycetacia and Verrucomicrobiae (Fig. 6 and Table SI9).

There was no correlation between the number of guts per sample and Shannon entropy per sample (Pearson correlation test between paired samples: *r* = 0.24, *t* = 1.38, df = 30, *p* = 0.17). There was a significant distinction between the gut microbiome of the four donor sets and the recipient *D. magna* measured by α- and β-diversity (α-diversity: *F* = 38.2; df = 1,18; *p* < 0.0001; β-diversity: *F* = 11.04, *R²* = 0.26, df = 1, *p* = 0.001; Figs. SI5 and SI6). In the donor populations there was a significant interaction between region and diet (*F* = 5.1 × 10^5^; df = 1, 1; *p* = 0.0008). In the population from Kortrijk the Shannon entropy was higher when they were fed with *M. aeruginosa* than when they were not fed with *M. aeruginosa*. In Leuven the opposite was true, there the Shannon entropy was higher when they were not fed with *M. aeruginosa* than when they were fed with *M. aeruginosa* (Fig. SI7). In the recipient *D. magna*, there was a significant three-way interaction between donor microbiome type, donor diet and genotype for the Shannon entropy (*F* = 131; df = 54; *p* = 0.0001, Table SI10 shows the results of the linear fixed-effects model with diet, microbiome type and genotype as fixed factors and genotype nested within pond and pond nested within region as random factor). Per donor diet, the interaction between microbiome type and recipient *D. magna* genotype was significant (pre-exposed to *M. aeruginosa*: *F* = 129; df = 5, 2; *p* = 0.007, not pre-exposed to *M. aeruginosa*: *F* = 174; df = 6, 2; *p* = 0.005). We observed inconsistent patterns on the genotype level. When the recipient *D. magna* received a donor microbiome in which the *D. magna* were pre-exposed to *M. aeruginosa*, three out of the eight genotypes (K_MS, L_OM2, and L_T8) had a lower Shannon entropy when they received a sympatric than an allopatric microbiome. For two genotypes (K_BH and K_KP) the sequencing partly failed making it impossible to compare the sympatric and allopatric treatments when the donor diet was pre-exposed to *M. aeruginosa*, and for the other three genotypes (K_ZWE2, L_T3 and L_T7) the Shannon entropy was higher when they received a sympatric than an allopatric microbiome. When the donor diet was not pre-exposed to *M. aeruginosa*, four out of the eight genotypes (K_ZWE2, L_OM2, L_T7 and L_T8) had a lower Shannon entropy when they received a sympatric than an allopatric microbiome, for one genotype (K_KP) there was no data and for the other three genotypes (K_BH, K_MS and L_T3) the Shannon entropy was higher when they received a sympatric than an allopatric microbiome (Fig. 4G, H and Fig. SI8).

The three-way interaction microbiome type × donor diet × genotype was not significant for the β-diversity on the total dataset, i.e., donors and recipients (Table SI11).

**Fig. 7:**
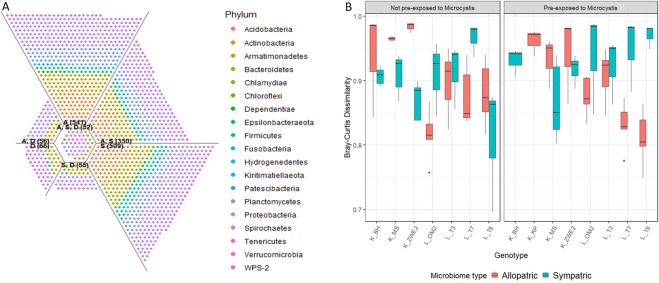
Unionplot and boxplot representing differences between donor and recipient gut microbiomes. **A** The Unionplot, representing the OTUs that are unique and shared between donor (D), allopatric (A) and sympatric (S) treatments. The numbers on the Unionplot represent the number of OTUs present in that compartment. **B** A boxplot, representing the Bray Curtis dissimilarity between donors and recipients across the treatments. The genotypes are grouped per donor diet (left and right panel) and microbiome type (red and blue color). The Bray Curtis dissimilarity varies less in the genotypes that received a sympatric microbiome pre-exposed to *M. aeruginosa*, than in the genotypes that received an allopatric or sympatric non pre-exposed to *M. aeruginosa* microbiome.

The Unionplot (Fig. 7A) showed that 71.2% of the OTUs from the donors were present in the recipients. 28.8% of the OTUs were unique for the donor population, 24.4% of the OTUs were shared between the donor and the allopatric treatments, 24% of the OTUs were shared between the donor and the sympatric treatments and 22.7% of the OTUs were shared between donor, allopatric and sympatric treatments. In addition, 10.4% of the OTUs in the recipients were also present in the donor samples. This was very similar in the allopatric (10.8%) and in the sympatric (11.1%) conditions. Analysis of the Bray Curtis dissimilarity matrix, to investigate the difference in distance between donor and recipients across the treatments, showed a significant three-way interaction between microbiome type, diet and genotype (*F* = 7.3, df = 2, *p* = 0.001; Table SI12). Investigation per microbiome type shows that in the allopatric treatments there was a main genotype effect (*F* = 5.05, df = 2, *p* = 0.01), reflecting strong variation in the microbial community between the recipient genotypes (Fig. 7B). In the sympatric treatments this genotype effect depended on the donor diet (diet × genotype: *F* = 6.5, df = 2, *p* = 0.003). In the sympatric treatments, there was a main genotype effect in *D. magna* that received a donor diet not pre-exposed to *M. aeruginosa* (*F* = 9.1, df = 2, *p* = 0.001). However, when they received a donor diet pre-exposed to *M. aeruginosa*, this genotype was no longer present (*F* = 0.8, df = 2, *p* = 0.45; Fig. 7B).

## Discussion

We here performed a reciprocal gut transplant experiment, in which germ-free *D. magna* were inoculated with a sympatric or an allopatric gut microbiome that was pre-exposed to toxic *M. aeruginosa* or not. All inoculated recipients were exposed to *M. aeruginosa* and the tested *Microcystis* strain was a neutral lab strain that has not been interacting with the *D. magna* genotypes in nature. We found strong *D. magna* donor diet (pre-exposed to *M. aeruginosa* or not) × donor microbiome type (sympatric vs. allopatric) × recipient genotype interactions for the life history traits survival and fecundity. Independent of the tested region, more than half of the genotypes showed a higher survival (seven out of nine) or fecundity (five out of eight) when they received a sympatric vs. an allopatric microbiome, especially if the donor microbiomes were pre-exposed to *M. aeruginosa*. This pattern where most *D. magna* genotypes had a higher fitness, in terms of survival and fecundity, when they received a local (sympatric) than a foreign (allopatric) microbiome, is in agreement with what we expected. It has earlier been shown that in stressful environments, hosts may benefit from locally adapted microbes, as hosts may use specific microbial communities with large phenotypic effects to specialize and persist in novel niches [37–41]. However, because of the reciprocal transplant procedure, our study is unique by providing a causal link between fitness effects and local microbiomes. Note, however, that we studied local adaptation *sensu lato*, given that the adaptation is not at the level of a particular population, but rather on the level of the region, reflecting regional adaptation. In future work it would be very interesting to investigate these effects further on a microscale within a region.

For body size there was also a significant donor diet × donor microbiome type × recipient genotype interaction, and the interactions between donor diet and donor microbiome type, and between donor microbiome type and recipient genotype were significant. Body size was smaller in *D. magna* with a donor microbiome that had been pre-exposed to *M. aeruginosa* than to the control diet and this was especially so for the sympatric microbiomes. We found a marginally significant negative correlation between the delta values of the donor microbiomes (*M. aeruginosa*—control treatment) for survival and body size in the recipient *D. magna* genotypes. This indicates that *D. magna* genotypes that had a higher survival with a microbiome pre-exposed to *M. aeruginosa* were smaller. This suggests that carrying a microbiome that is associated with a higher tolerance upon *M. aeruginosa* exposure comes with a cost in terms of body size. A trade-off between growth and survival is shown in many organisms [72–74]. Here we show that this trade-off in *Daphnia* may be mediated through the microbiome, as also suggested by Callens et al. [58]. Hosts with particular microbiomes apparently divide energy towards growth or longevity, but not to the two traits simultaneously: they (1) grow slower with higher tolerance to stressful conditions, or (2) they grow faster without a higher tolerance [74].

There was a significant difference in α- and β-diversity in the donor vs. the recipient microbiomes. The donor microbiomes consisted mainly of Gammaproteobacteria and Bacteroida (Flavobacteriia). In the study of Macke et al. [42], *D. magna* genotypes that were tolerant to *Microcystis*, contained more Flavobacteriia. Gammaproteobacteria and Flavobacteriia have been described to be part of the mainly aerobic *D. magna* gut bacterial community [75]. Gammaproteobacteria typically break down and ferment complex sugars and provide particularly important digestive roles [40, 76]. Members of the Flavobacteriia group cause the lysis of *Microcystis* cells and degrade dissolved organic matter derived from intracellular products of *Microcystis* [77, 78]. The presence of Flavobacteriia in the gut of *Daphnia* may therefore provide the host individual access to otherwise inaccessible nutrients in addition to detoxification of cyanotoxins. The recipient gut microbiomes were more diverse than the donor gut microbiomes. Next to Gammaproteobacteria and Bacteroida, the recipient gut microbiomes also contained bacteria from the following classes: Actinobacteria, Alphaproteobacteria, Bacilli, Planctomycetacia and Verrucomicrobiae. The recipient microbiomes were inoculated with the donor microbiomes for 48 h in sterile-filtered tap water in our transplant experiment. After these 48 h, the recipient *D. magna* were placed in a mixture of pond water from the region of origin, which may explain the relatively higher diversity and the presence of environmental bacteria in the gut microbiome of the recipient [79] *D. magna*. According to the Unionplot, 71.2% of the OTUs were shared between donors and recipients, showing that in the 48 h the recipients took up a substantial part of the bacteria from the pooled donor inoculum. The analysis of the Bray Curtis dissimilarity matrix, showed that *D. magna* that received a sympatric microbiome that was pre-exposed to *M. aeruginosa* had a more convergent microbiome across the genotypes. This was demonstrated by the distance between donors and recipients varying less across the genotypes in the sympatric treatments that were pre-exposed to *M. aeruginosa*, than across the genotypes in allopatric and sympatric treatments not pre-exposed to *M. aeruginosa* (Fig. 7B).

The host *D. magna* may follow two patterns with respect to the selective uptake of microbial strains to obtain an increased tolerance upon toxic cyanobacterial exposure: (1) selection of a few adapted strains, or (2) selection for a high diversity of strains with complementary gene functions (Fig. 1). In this study, we found support for both patterns. Five of the eight genotypes (K_BH, K_MS, L_OM2, L_T7 andL_T8) had higher fitness traits (survival and/or fecundity). In three of these recipient genotypes (K_MS, L_OM2, L_T8) there was a lower Shannon entropy in the sympatric vs. allopatric donor microbiome when the donors were pre-exposed to *M. aeruginosa*. This suggests a selective uptake of a microbiome with beneficial functions, especially in stressful environments. This resembles to patterns observed in plants that recruit their microbiome in a host species-specific way and whereby the plant genotype affects the accumulation of micro-organisms that help the plant to defend itself, e.g., against pathogen attacks [80–82]. Selection of specific OTUs by these *D. magna* genotypes may lead to a lower chance of establishing other OTUs. This can be enhanced by priority effects of the first establishing bacterial strains leaving less space for other bacteria from the environment to enter and settle. These bacteria by interacting strongly with the host, will be strong competitors, and outcompete other incoming bacteria. In turn, this may then affect the host phenotype. However, two out of the eight genotypes (K_ZWE and L_T3) followed the second pattern, where both *D. magna* fitness (survival and/or fecundity) and bacterial diversity were higher in the sympatric than in the allopatric treatment. Hosts can also benefit from a more diverse microbiome. For example, it has been shown that mice with a natural, more diverse, microbiome had a higher fitness and a lower inflammation response to two diseases than mice with a laboratory, less diverse, microbiome [45]. It is possible that a more diverse community contains a wider array of metabolic capabilities, allowing for example a better growth [83]. Alternatively, the second pattern can also be explained through *Daphnia* genotypes being non-selective and picking up randomly a high diversity of (also non-adaptive) strains in stressful environments.

We conclude that most *D. magna* genotypes had a higher fitness in terms of survival and fecundity, when they received a sympatric than an allopatric microbiome. These fitness benefits from a local microbiome were, however, genotype dependent, which conforms the hypothesis that different *Daphnia* genotypes differ in their tolerance to *M. aeruginosa* [42] and different genotypes select different microbiomes [35]. As such the microbiome can help in structuring the wider freshwater bacterioplankton community through microbiome mediated eco-evolutionary dynamics [43, 84]. In some *D. magna* genotypes fitness was higher while bacterial diversity was lower for sympatric than for allopatric microbiomes, suggesting selection of certain microbiome strains. While in others both the fitness and bacterial diversity were higher in sympatric than allopatric treatments, suggesting selection of a high-bacterial diversity. Future research should confirm if particular adaptive strains interact with the host genotype to structure the microbial community in the *Daphnia* gut in stressful cyanobacterial environments.

## Supplementary information

Table SI1

Table SI2

Table SI3

Table SI4

Table SI5

Table SI6

Table SI7

Table SI8

Table SI9

Table SI10

Table SI11

Table SI12

Figure SI1

Figure SI2

Figure SI3

Figure SI4

Figure SI5

figure SI6

Figure SI7

Figure SI8

## Data Availability

The datasets generated for this study can be found in the NCBI, under accession number PRJNA690081.
