# Supplementary material for: Locally adapted gut microbiomes mediate host stress tolerance
Source: ISME J. 2021 Mar 3;15(8):2401–14. doi: 10.1038/s41396-021-00940-y (PMC8319338; doi:10.1038/s41396-021-00940-y)
Supplement: Supplementary file 1 — Table SI1 [file 41396_2021_940_MOESM1_ESM.docx]

Table SI1

| **Genotype** | **Pond** | **Coordinates** |
| --- | --- | --- |
| **Kortrijk** |  |  |
| K_BH | Blauwe Hoeve (K1) | 50°48’58.63”N, 3°16’17.28”E |
| K_KP | Kennedypark (K2) | 50°48’60.50”N, 3°16’38.45”E |
| K_MS | Morinnestraat (K3) | 50°48’20.83”N, 3°18’44.91”E |
| K_ZWE1 | Zwevegem (K5) | 50°49’4.37”N, 3°20’17.37”E |
| K_ZWE2 | Zwevegem (K5) | 50°49’4.37”N, 3°20’17.37”E |
| **Leuven** |  |  |
| L_OM2 | Heverlee, Abdij van ’t Park (L1) | 50°51’45.0”N, 04°42’58.8”E |
| L_T2 | Oud Heverlee (L2) | 50°50’24.0”N, 04°39’40.4”E |
| L_T3 | Oud Heverlee (L2) | 50°50’24.0”N, 04°39’40.4”E |
| L_T7 | Oud Heverlee (L2) | 50°50’24.0”N, 04°39’40.4”E |
| L_T8 | Oud Heverlee (L2) | 50°50’24.0”N, 04°39’40.4”E |
