# Supplementary material for: Locally adapted gut microbiomes mediate host stress tolerance
Source: ISME J. 2021 Mar 3;15(8):2401–14. doi: 10.1038/s41396-021-00940-y (PMC8319338; doi:10.1038/s41396-021-00940-y)
Supplement: Supplementary file 2 — Table SI2 [file 41396_2021_940_MOESM2_ESM.docx]

Table SI2

| Genotype | Received a sympatric donor microbiome pre-exposed to *M. aeruginosa* | Received a sympatric donor microbiome not pre-exposed to *M. aeruginosa* | Received an allopatric donor microbiome pre-exposed to *M. aeruginosa* | Received an allopatric donor microbiome not pre-exposed to *M. aeruginosa* |
| --- | --- | --- | --- | --- |
| K_BH | 3 | 4 | 4 | 2 |
| K_KP | 1 | 2 | 2 | 1 |
| K_MS | 3 | 3 | 4 | 5 |
| K_ZWE2 | 2 | 2 | 1 | 2 |
| L_OM2 | 2 | 3 | 2 | 3 |
| L_T3 | 6 | 3 | 3 | 5 |
| L_T7 | 3 | 3 | 3 | 2 |
| L_T8 | 3 | 2 | 5 | 5 |
