# Supplementary material for: Locally adapted gut microbiomes mediate host stress tolerance
Source: ISME J. 2021 Mar 3;15(8):2401–14. doi: 10.1038/s41396-021-00940-y (PMC8319338; doi:10.1038/s41396-021-00940-y)
Supplement: Supplementary file 3 — Table SI3 [file 41396_2021_940_MOESM3_ESM.docx]

Table SI3

| **Model: GLMER with random factor (1\|Region:Pond) on percentage survived *Daphnia*** | **df** | **AIC** |
| --- | --- | --- |
| fit1 <- glmer(PercentSurvived~Diet*MicrobiomeType*Region*Pond*Genotype + (1\|Region:Pond:Genotype) , data=dataS1) | 38 | 679.5210 |
| fit2 <- glmer(PercentSurvived~Diet*MicrobiomeType*Region*Pond + (1\|Region:Pond:Genotype) , data=dataS1) | 26 | 778.4938 |
| fit3 <- glmer(PercentSurvived~Diet*MicrobiomeType*Region*Genotype + (1\|Region:Pond:Genotype) , data=dataS1) | 38 | 679.5210 |
| fit4 <- glmer(PercentSurvived~Diet*MicrobiomeType*Pond*Genotype + (1\|Region:Pond:Genotype) , data=dataS1) | 38 | 676.7484 |
| fit5 <- glmer(PercentSurvived~Diet*Region*Pond*Genotype + (1\|Region:Pond:Genotype) , data=dataS1) | 38 | 676.7484 |
| fit6 <- glmer(PercentSurvived~MicrobiomeType*Region*Pond*Genotype + (1\|Region:Pond:Genotype) , data=dataS1) | 20 | 814.7191 |
| fit7 <- glmer(PercentSurvived~Diet*MicrobiomeType*Region + (1\|Region:Pond:Genotype) , data=dataS1) | 10 | 900.2830 |
| fit8 <- glmer(PercentSurvived~Diet*MicrobiomeType*Pond + (1\|Region:Pond:Genotype) , data=dataS1) | 26 | 775.7212 |
| fit9 <- glmer(PercentSurvived~Diet*Region*Pond + (1\|Region:Pond:Genotype) , data=dataS1) | 26 | 775.7212 |
| fit10 <- glmer(PercentSurvived~MicrobiomeType*Region*Pond + (1\|Region:Pond:Genotype) , data=dataS1) | 14 | 866.2487 |
| fit11<- glmer(PercentSurvived~Diet*MicrobiomeType*Genotype + (1\|Region:Pond:Genotype) , data=dataS1) | 38 | 676.7484 |
| fit12<- glmer(PercentSurvived~Diet*Region*Genotype + (1\|Region:Pond:Genotype) , data=dataS1) | 38 | 676.7484 |
| fit13<- glmer(PercentSurvived~MicrobiomeType*Region*Genotype + (1\|Region:Pond:Genotype) , data=dataS1) | 20 | 814.7191 |
| fit14<- glmer(PercentSurvived~Diet*Pond*Genotype + (1\|Region:Pond:Genotype) , data=dataS1) | 20 | 813.9995 |
| fit15<- glmer(PercentSurvived~MicrobiomeType*Pond*Genotype + (1\|Region:Pond:Genotype) , data=dataS1) | 20 | 813.3328 |
| fit16<- glmer(PercentSurvived~Region*Pond*Genotype + (1\|Region:Pond:Genotype) , data=dataS1) | 20 | 813.3328 |
| fit17 <- glmer(PercentSurvived~Diet*MicrobiomeType + (1\|Region:Pond:Genotype) , data=dataS1) | 6 | 925.7001 |
| fit18 <- glmer(PercentSurvived~Diet*Region + (1\|Region:Pond:Genotype) , data=dataS1) | 6 | 926.0586 |
| fit19 <- glmer(PercentSurvived~Diet*Pond + (1\|Region:Pond:Genotype) , data=dataS1) | 14 | 870.6112 |
| fit20 <- glmer(PercentSurvived~Diet*Genotype + (1\|Region:Pond:Genotype) , data=dataS1) | 20 | 813.995 |
| fit21 <- glmer(PercentSurvived~MicrobiomeType*Region + (1\|Region:Pond:Genotype) , data=dataS1) | 6 | 924.4231 |
| fit22 <- glmer(PercentSurvived~MicrobiomeType*Pond + (1\|Region:Pond:Genotype) , data=dataS1) | 14 | 864.8624 |
| fit23 <- glmer(PercentSurvived~MicrobiomeType*Genotype + (1\|Region:Pond:Genotype) , data=dataS1) | 20 | 813.3328 |
| fit24 <- glmer(PercentSurvived~Region*Pond + (1\|Region:Pond:Genotype) , data=dataS1) | 14 | 864.8624 |
| fit25 <- glmer(PercentSurvived~Region*Genotype + (1\|Region:Pond:Genotype) , data=dataS1) | 20 | 813.3328 |
| fit26 <- glmer(PercentSurvived~Pond*Genotype + (1\|Region:Pond:Genotype) , data=dataS1) | 11 | 872.3569 |
| fit27 <- glmer(PercentSurvived~Diet + (1\|Region:Pond:Genotype) , data=dataS1) | 4 | 937.5637 |
| fit28 <- glmer(PercentSurvived~MicrobiomeType + (1\|Region:Pond:Genotype) , data=dataS1) | 4 | 936.4223 |
| fit29 <- glmer(PercentSurvived~Region + (1\|Region:Pond:Genotype) , data=dataS1) | 4 | 936.7077 |
| fit30 <- glmer(PercentSurvived~Pond + (1\|Region:Pond:Genotype) , data=dataS1) | 8 | 908.9325 |
| fit31 <- glmer(PercentSurvived~Genotype + (1\|Region:Pond:Genotype) , data=dataS1) | 11 | 872.3569 |
| **Model: GLMER with random factor (1\|Region:Pond) on percentage survived *Daphnia*** | **df** | **AIC** |
| fit1 <- glmer(PercentSurvived~Diet*MicrobiomeType*Region*Pond*Genotype + (1\|Region:Pond) , data=dataS1) | 38 | 679.5210 |
| fit2 <- glmer(PercentSurvived~Diet*MicrobiomeType*Region*Pond + (1\|Region:Pond) , data=dataS1) | 26 | 778.0628 |
| fit3 <- glmer(PercentSurvived~Diet*MicrobiomeType*Region*Genotype + (1\|Region:Pond) , data=dataS1) | 38 | 679.5210 |
| fit4 <- glmer(PercentSurvived~Diet*MicrobiomeType*Pond*Genotype + (1\|Region:Pond) , data=dataS1) | 38 | 676.7484 |
| fit5 <- glmer(PercentSurvived~Diet*Region*Pond*Genotype + (1\|Region:Pond) , data=dataS1) | 38 | 676.7484 |
| fit6 <- glmer(PercentSurvived~MicrobiomeType*Region*Pond*Genotype + (1\|Region:Pond) , data=dataS1) | 20 | 814.7191 |
| fit7 <- glmer(PercentSurvived~Diet*MicrobiomeType*Region + (1\|Region:Pond) , data=dataS1) | 10 | 905.8093 |
| fit8 <- glmer(PercentSurvived~Diet*MicrobiomeType*Pond + (1\|Region:Pond) , data=dataS1) | 26 | 784.2902 |
| fit9 <- glmer(PercentSurvived~Diet*Region*Pond + (1\|Region:Pond) , data=dataS1) | 26 | 784.2902 |
| fit10 <- glmer(PercentSurvived~MicrobiomeType*Region*Pond + (1\|Region:Pond) , data=dataS1) | 14 | 876.9831 |
| fit11<- glmer(PercentSurvived~Diet*MicrobiomeType*Genotype + (1\|Region:Pond) , data=dataS1) | 38 | 676.7484 |
| fit12<- glmer(PercentSurvived~Diet*Region*Genotype + (1\|Region:Pond) , data=dataS1) | 38 | 676.7484 |
| fit13<- glmer(PercentSurvived~MicrobiomeType*Region*Genotype + (1\|Region:Pond) , data=dataS1) | 20 | 814.7191 |
| fit14<- glmer(PercentSurvived~Diet*Pond*Genotype + (1\|Region:Pond) , data=dataS1) | 20 | 813.9995 |
| fit15<- glmer(PercentSurvived~MicrobiomeType*Pond*Genotype + (1\|Region:Pond) , data=dataS1) | 20 | 813.3328 |
| fit16<- glmer(PercentSurvived~Region*Pond*Genotype + (1\|Region:Pond) , data=dataS1) | 20 | 813.3328 |
| fit17 <- glmer(PercentSurvived~Diet*MicrobiomeType + (1\|Region:Pond) , data=dataS1) | 6 | 930.3198 |
| fit18 <- glmer(PercentSurvived~Diet*Region + (1\|Region:Pond) , data=dataS1) | 6 | 930.8629 |
| fit19 <- glmer(PercentSurvived~Diet*Pond + (1\|Region:Pond) , data=dataS1) | 14 | 875.2823 |
| fit20 <- glmer(PercentSurvived~Diet*Genotype + (1\|Region:Pond) , data=dataS1) | 20 | 813.9995 |
| fit21 <- glmer(PercentSurvived~MicrobiomeType*Region + (1\|Region:Pond) , data=dataS1) | 6 | 930.9567 |
| fit22 <- glmer(PercentSurvived~MicrobiomeType*Pond + (1\|Region:Pond) , data=dataS1) | 14 | 875.5968 |
| fit23 <- glmer(PercentSurvived~MicrobiomeType*Genotype + (1\|Region:Pond) , data=dataS1) | 20 | 813.3328 |
| fit24 <- glmer(PercentSurvived~Region*Pond + (1\|Region:Pond) , data=dataS1) | 14 | 875.5968 |
| fit25 <- glmer(PercentSurvived~Region*Genotype + (1\|Region:Pond) , data=dataS1) | 20 | 813.3328 |
| fit26 <- glmer(PercentSurvived~Pond*Genotype + (1\|Region:Pond) , data=dataS1) | 11 | 872.3569 |
| fit27 <- glmer(PercentSurvived~Diet + (1\|Region:Pond) , data=dataS1) | 4 | 941.9731 |
| fit28 <- glmer(PercentSurvived~MicrobiomeType + (1\|Region:Pond) , data=dataS1) | 4 | 941.5949 |
| fit29 <- glmer(PercentSurvived~Region + (1\|Region:Pond) , data=dataS1) | 4 | 941.9838 |
| fit30 <- glmer(PercentSurvived~Pond + (1\|Region:Pond) , data=dataS1) | 8 | 914.5121 |
| fit31 <- glmer(PercentSurvived~Genotype + (1\|Region:Pond) , data=dataS1) | 11 | 872.3569 |
| **Model: LMER with random factor (1\|Region:Pond:Genotype) on percentage survived *Daphnia*** | **df** | **AIC** |
| fit1 <- lmer(PercentSurvived~Diet*MicrobiomeType*Region*Pond*Genotype + (1\|Region:Pond:Genotype) , data=dataS1) | 38 | 679.5210 |
| fit2 <- lmer(PercentSurvived~Diet*MicrobiomeType*Region*Pond + (1\|Region:Pond:Genotype) , data=dataS1) | 26 | 778.4938 |
| fit3 <- lmer(PercentSurvived~Diet*MicrobiomeType*Region*Genotype + (1\|Region:Pond:Genotype) , data=dataS1) | 38 | 679.5210 |
| fit4 <- lmer(PercentSurvived~Diet*MicrobiomeType*Pond*Genotype + (1\|Region:Pond:Genotype) , data=dataS1) | 38 | 676.7484 |
| fit5 <- lmer(PercentSurvived~Diet*Region*Pond*Genotype + (1\|Region:Pond:Genotype) , data=dataS1) | 38 | 676.7484 |
| fit6 <- lmer(PercentSurvived~MicrobiomeType*Region*Pond*Genotype + (1\|Region:Pond:Genotype) , data=dataS1) | 20 | 814.7191 |
| fit7 <- lmer(PercentSurvived~Diet*MicrobiomeType*Region + (1\|Region:Pond:Genotype) , data=dataS1) | 10 | 900.2830 |
| fit8 <- lmer(PercentSurvived~Diet*MicrobiomeType*Pond + (1\|Region:Pond:Genotype) , data=dataS1) | 26 | 775.7212 |
| fit9 <- lmer(PercentSurvived~Diet*Region*Pond + (1\|Region:Pond:Genotype) , data=dataS1) | 26 | 775.7212 |
| fit10 <- lmer(PercentSurvived~MicrobiomeType*Region*Pond + (1\|Region:Pond:Genotype) , data=dataS1) | 14 | 866.2487 |
| fit11<- lmer(PercentSurvived~Diet*MicrobiomeType*Genotype + (1\|Region:Pond:Genotype) , data=dataS1) | 38 | 676.7484 |
| fit12<- lmer(PercentSurvived~Diet*Region*Genotype + (1\|Region:Pond:Genotype) , data=dataS1) | 38 | 676.7484 |
| fit13<- lmer(PercentSurvived~MicrobiomeType*Region*Genotype + (1\|Region:Pond:Genotype) , data=dataS1) | 20 | 814.7191 |
| fit14<- lmer(PercentSurvived~Diet*Pond*Genotype + (1\|Region:Pond:Genotype) , data=dataS1) | 20 | 813.9995 |
| fit15<- lmer(PercentSurvived~MicrobiomeType*Pond*Genotype + (1\|Region:Pond:Genotype) , data=dataS1) | 20 | 813.3328 |
| fit16<- lmer(PercentSurvived~Region*Pond*Genotype + (1\|Region:Pond:Genotype) , data=dataS1) | 20 | 813.3328 |
| fit17 <- lmer(PercentSurvived~Diet*MicrobiomeType + (1\|Region:Pond:Genotype) , data=dataS1) | 6 | 925.7001 |
| fit18 <- lmer(PercentSurvived~Diet*Region + (1\|Region:Pond:Genotype) , data=dataS1) | 6 | 926.0586 |
| fit19 <- lmer(PercentSurvived~Diet*Pond + (1\|Region:Pond:Genotype) , data=dataS1) | 14 | 870.6112 |
| fit20 <- lmer(PercentSurvived~Diet*Genotype + (1\|Region:Pond:Genotype) , data=dataS1) | 20 | 813.9995 |
| fit21 <- lmer(PercentSurvived~MicrobiomeType*Region + (1\|Region:Pond:Genotype) , data=dataS1) | 6 | 924.4231 |
| fit22 <- lmer(PercentSurvived~MicrobiomeType*Pond + (1\|Region:Pond:Genotype) , data=dataS1) | 14 | 864.8624 |
| fit23 <- lmer(PercentSurvived~MicrobiomeType*Genotype + (1\|Region:Pond:Genotype) , data=dataS1) | 20 | 813.3328 |
| fit24 <- lmer(PercentSurvived~Region*Pond + (1\|Region:Pond:Genotype) , data=dataS1) | 14 | 864.8624 |
| fit25 <- lmer(PercentSurvived~Region*Genotype + (1\|Region:Pond:Genotype) , data=dataS1) | 20 | 813.3328 |
| fit26 <- lmer(PercentSurvived~Pond*Genotype + (1\|Region:Pond:Genotype) , data=dataS1) | 11 | 872.3569 |
| fit27 <- lmer(PercentSurvived~Diet + (1\|Region:Pond:Genotype) , data=dataS1) | 4 | 937.5637 |
| fit28 <- lmer(PercentSurvived~MicrobiomeType + (1\|Region:Pond:Genotype) , data=dataS1) | 4 | 936.4223 |
| fit29 <- lmer(PercentSurvived~Region + (1\|Region:Pond:Genotype) , data=dataS1) | 4 | 936.7077 |
| fit30 <- lmer(PercentSurvived~Pond + (1\|Region:Pond:Genotype) , data=dataS1) | 8 | 908.9325 |
| fit31 <- lmer(PercentSurvived~Genotype + (1\|Region:Pond:Genotype) , data=dataS1) | 11 | 872.3569 |
| **Model: LMER with random factor (1\|Region:Pond) on percentage survived *Daphnia*** | **df** | **AIC** |
| fit1 <- lmer(PercentSurvived~Diet*MicrobiomeType*Region*Pond*Genotype + (1\|Region:Pond) , data=dataS1) | 38 | 679.5210 |
| fit2 <- lmer(PercentSurvived~Diet*MicrobiomeType*Region*Pond + (1\|Region:Pond) , data=dataS1) | 26 | 787.0628 |
| fit3 <- lmer(PercentSurvived~Diet*MicrobiomeType*Region*Genotype + (1\|Region:Pond) , data=dataS1) | 38 | 679.5210 |
| fit4 <- lmer(PercentSurvived~Diet*MicrobiomeType*Pond*Genotype + (1\|Region:Pond) , data=dataS1) | 38 | 676.7484 |
| fit5 <- lmer(PercentSurvived~Diet*Region*Pond*Genotype + (1\|Region:Pond) , data=dataS1) | 38 | 676.7484 |
| fit6 <- lmer(PercentSurvived~MicrobiomeType*Region*Pond*Genotype + (1\|Region:Pond) , data=dataS1) | 20 | 814.7191 |
| fit7 <- lmer(PercentSurvived~Diet*MicrobiomeType*Region + (1\|Region:Pond) , data=dataS1) | 10 | 905.8093 |
| fit8 <- lmer(PercentSurvived~Diet*MicrobiomeType*Pond + (1\|Region:Pond) , data=dataS1) | 26 | 784.2902 |
| fit9 <- lmer(PercentSurvived~Diet*Region*Pond + (1\|Region:Pond) , data=dataS1) | 26 | 784.2902 |
| fit10 <- lmer(PercentSurvived~MicrobiomeType*Region*Pond + (1\|Region:Pond) , data=dataS1) | 14 | 876.9831 |
| fit11<- lmer(PercentSurvived~Diet*MicrobiomeType*Genotype + (1\|Region:Pond) , data=dataS1) | 38 | 676.7484 |
| fit12<- lmer(PercentSurvived~Diet*Region*Genotype + (1\|Region:Pond) , data=dataS1) | 38 | 676.7484 |
| fit13<- lmer(PercentSurvived~MicrobiomeType*Region*Genotype + (1\|Region:Pond) , data=dataS1) | 20 | 814.7191 |
| fit14<- lmer(PercentSurvived~Diet*Pond*Genotype + (1\|Region:Pond) , data=dataS1) | 20 | 813.9995 |
| fit15<- lmer(PercentSurvived~MicrobiomeType*Pond*Genotype + (1\|Region:Pond) , data=dataS1) | 20 | 813.3328 |
| fit16<- lmer(PercentSurvived~Region*Pond*Genotype + (1\|Region:Pond) , data=dataS1) | 20 | 813.3328 |
| fit17 <- lmer(PercentSurvived~Diet*MicrobiomeType + (1\|Region:Pond) , data=dataS1) | 6 | 930.3198 |
| fit18 <- lmer(PercentSurvived~Diet*Region + (1\|Region:Pond) , data=dataS1) | 6 | 930.8629 |
| fit19 <- lmer(PercentSurvived~Diet*Pond + (1\|Region:Pond) , data=dataS1) | 14 | 875.2823 |
| fit20 <- lmer(PercentSurvived~Diet*Genotype + (1\|Region:Pond) , data=dataS1) | 20 | 813.9995 |
| fit21 <- lmer(PercentSurvived~MicrobiomeType*Region + (1\|Region:Pond) , data=dataS1) | 6 | 930.9567 |
| fit22 <- lmer(PercentSurvived~MicrobiomeType*Pond + (1\|Region:Pond) , data=dataS1) | 14 | 875.5968 |
| fit23 <- lmer(PercentSurvived~MicrobiomeType*Genotype + (1\|Region:Pond) , data=dataS1) | 20 | 813.3328 |
| fit24 <- lmer(PercentSurvived~Region*Pond + (1\|Region:Pond) , data=dataS1) | 14 | 875.5968 |
| fit25 <- lmer(PercentSurvived~Region*Genotype + (1\|Region:Pond) , data=dataS1) | 20 | 813.3328 |
| fit26 <- lmer(PercentSurvived~Pond*Genotype + (1\|Region:Pond) , data=dataS1) | 11 | 872.3569 |
| fit27 <- lmer(PercentSurvived~Diet + (1\|Region:Pond) , data=dataS1) | 4 | 941.9731 |
| fit28 <- lmer(PercentSurvived~MicrobiomeType + (1\|Region:Pond) , data=dataS1) | 4 | 941.5949 |
| fit29 <- lmer(PercentSurvived~Region + (1\|Region:Pond) , data=dataS1) | 4 | 941.9838 |
| fit30 <- lmer(PercentSurvived~Pond + (1\|Region:Pond) , data=dataS1) | 8 | 914.5121 |
| fit31 <- lmer(PercentSurvived~Genotype + (1\|Region:Pond) , data=dataS1) | 11 | 872.3569 |
| **Model: GLM on percentage survived *Daphnia*** | **df** | **AIC** |
| fit1 <- glm(PercentSurvived~Diet*MicrobiomeType*Region*Pond*Genotype, data=dataS1) | 37 | 963.3175 |
| fit2 <- glm(PercentSurvived~Diet*MicrobiomeType*Region*Pond, data=dataS1) | 25 | 978.3827 |
| fit3 <- glm(PercentSurvived~Diet*MicrobiomeType*Region*Genotype data=dataS1) | 37 | 963.3175 |
| fit4 <- glm(PercentSurvived~Diet*MicrobiomeType*Pond*Genotype, data=dataS1) | 37 | 963.3175 |
| fit5 <- glm(PercentSurvived~Diet*Region*Pond*Genotype, data=dataS1) | 37 | 963.3175 |
| fit6 <- glm(PercentSurvived~MicrobiomeType*Region*Pond*Genotype, data=dataS1) | 19 | 944.0911 |
| fit7 <- glm(PercentSurvived~Diet*MicrobiomeType*Region, data=dataS1) | 9 | 958.5865 |
| fit8 <- glm(PercentSurvived~Diet*MicrobiomeType*Pond, data=dataS1) | 25 | 978.3827 |
| fit9 <- glm(PercentSurvived~Diet*Region*Pond, data=dataS1) | 25 | 978.3827 |
| fit10 <- glm(PercentSurvived~MicrobiomeType*Region*Pond, data=dataS1) | 13 | 963.5784 |
| fit11<- glm(PercentSurvived~Diet*MicrobiomeType*Genotype, data=dataS1) | 37 | 963.3175 |
| fit12<- glm(PercentSurvived~Diet*Region*Genotype, data=dataS1) | 37 | 963.3175 |
| fit13<- glm(PercentSurvived~MicrobiomeType*Region*Genotype, data=dataS1) | 19 | 944.0911 |
| fit14<- glm(PercentSurvived~Diet*Pond*Genotype, data=dataS1) | 19 | 944.8397 |
| fit15<- glm(PercentSurvived~MicrobiomeType*Pond*Genotype, data=dataS1) | 19 | 944.0911 |
| fit16<- glm(PercentSurvived~Region*Pond*Genotype, data=dataS1) | 19 | 944.0911 |
| fit17 <- glm(PercentSurvived~Diet*MicrobiomeType, data=dataS1) | 5 | 952.8640 |
| fit18 <- glm(PercentSurvived~Diet*Region, data=dataS1) | 5 | 953.4297 |
| fit19 <- glm(PercentSurvived~Diet*Pond, data=dataS1) | 13 | 963.2143 |
| fit20 <- glm(PercentSurvived~Diet*Genotype, data=dataS1) | 19 | 944.0911 |
| fit21 <- glm(PercentSurvived~MicrobiomeType*Region, data=dataS1) | 5 | 953.6085 |
| fit22 <- glm(PercentSurvived~MicrobiomeType*Pond, data=dataS1) | 13 | 963.5784 |
| fit23 <- glm(PercentSurvived~MicrobiomeType*Genotype, data=dataS1) | 19 | 944.0911 |
| fit24 <- glm(PercentSurvived~Region*Pond, data=dataS1) | 13 | 963.5784 |
| fit25 <- glm(PercentSurvived~Region*Genotype, data=dataS1) | 19 | 944.0911 |
| fit26 <- glm(PercentSurvived~Pond*Genotype, data=dataS1) | 10 | 930.3863 |
| fit27 <- glm(PercentSurvived~Diet, data=dataS1) | 3 | 950.8786 |
| fit28 <- glm(PercentSurvived~MicrobiomeType, data=dataS1) | 3 | 950.4919 |
| fit29 <- glm(PercentSurvived~Region, data=dataS1) | 3 | 950.8899 |
| fit30 <- glm(PercentSurvived~Pond, data=dataS1) | 7 | 953.2309 |
| fit31 <- glm(PercentSurvived~Genotype, data=dataS1) | 10 | 930.3863 |
| **Model: LMER with random factor (1\|Region:Pond:Genotype) on total number of juveniles** | **df** | **AIC** |
| fit1 <- lmer(Juveniles~Diet*MicrobiomeType*Region*Pond*Genotype + (1\|Region:Pond:Genotype) , data=dataN1) | 34 | 567.8499 |
| fit2 <- lmer(Juveniles~Diet*MicrobiomeType*Region*Pond + (1\|Region:Pond:Genotype) , data=dataN1) | 26 | 607.2696 |
| fit3 <- lmer(Juveniles~Diet*MicrobiomeType*Region*Genotype + (1\|Region:Pond:Genotype) , data=dataN1) | 34 | 567.8499 |
| fit4 <- lmer(Juveniles~Diet*MicrobiomeType*Pond*Genotype + (1\|Region:Pond:Genotype) , data=dataN1) | 34 | 565.0774 |
| fit5 <- lmer(Juveniles~Diet*Region*Pond*Genotype + (1\|Region:Pond:Genotype) , data=dataN1) | 34 | 565.0774 |
| fit6 <- lmer(Juveniles~MicrobiomeType*Region*Pond*Genotype + (1\|Region:Pond:Genotype) , data=dataN1) | 18 | 660.8931 |
| fit7 <- lmer(Juveniles~Diet*MicrobiomeType*Region + (1\|Region:Pond:Genotype) , data=dataN1) | 10 | 702.3937 |
| fit8 <- lmer(Juveniles~Diet*MicrobiomeType*Pond + (1\|Region:Pond:Genotype) , data=dataN1) | 26 | 604.4970 |
| fit9 <- lmer(Juveniles~Diet*Region*Pond + (1\|Region:Pond:Genotype) , data=dataN1) | 26 | 604.4970 |
| fit10 <- lmer(Juveniles~MicrobiomeType*Region*Pond + (1\|Region:Pond:Genotype) , data=dataN1) | 14 | 679.1022 |
| fit11<- lmer(Juveniles~Diet*MicrobiomeType*Genotype + (1\|Region:Pond:Genotype) , data=dataN1) | 34 | 565.0774 |
| fit12<- lmer(Juveniles~Diet*Region*Genotype + (1\|Region:Pond:Genotype) , data=dataN1) | 34 | 565.0774 |
| fit13<- lmer(Juveniles~MicrobiomeType*Region*Genotype + (1\|Region:Pond:Genotype) , data=dataN1) | 18 | 660.8931 |
| fit14<- lmer(Juveniles~Diet*Pond*Genotype + (1\|Region:Pond:Genotype) , data=dataN1) | 18 | 660.7503 |
| fit15<- lmer(Juveniles~MicrobiomeType*Pond*Genotype + (1\|Region:Pond:Genotype) , data=dataN1) | 18 | 659.5068 |
| fit16<- lmer(Juveniles~Region*Pond*Genotype + (1\|Region:Pond:Genotype) , data=dataN1) | 18 | 659.5068 |
| fit17 <- lmer(Juveniles~Diet*MicrobiomeType + (1\|Region:Pond:Genotype) , data=dataN1) | 6 | 722.0320 |
| fit18 <- lmer(Juveniles~Diet*Region + (1\|Region:Pond:Genotype) , data=dataN1) | 6 | 723.6910 |
| fit19 <- lmer(Juveniles~Diet*Pond + (1\|Region:Pond:Genotype) , data=dataN1) | 14 | 677.9053 |
| fit20 <- lmer(Juveniles~Diet*Genotype + (1\|Region:Pond:Genotype) , data=dataN1) | 18 | 660.7503 |
| fit21 <- lmer(Juveniles~MicrobiomeType*Region + (1\|Region:Pond:Genotype) , data=dataN1) | 6 | 720.0995 |
| fit22 <- lmer(Juveniles~MicrobiomeType*Pond + (1\|Region:Pond:Genotype) , data=dataN1) | 14 | 677.7159 |
| fit23 <- lmer(Juveniles~MicrobiomeType*Genotype + (1\|Region:Pond:Genotype) , data=dataN1) | 18 | 659.5068 |
| fit24 <- lmer(Juveniles~Region*Pond + (1\|Region:Pond:Genotype) , data=dataN1) | 14 | 677.7159 |
| fit25 <- lmer(Juveniles~Region*Genotype + (1\|Region:Pond:Genotype) , data=dataN1) | 18 | 659.5068 |
| fit26 <- lmer(Juveniles~Pond*Genotype + (1\|Region:Pond:Genotype) , data=dataN1) | 10 | 693.0774 |
| fit27 <- lmer(Juveniles~Diet + (1\|Region:Pond:Genotype) , data=dataN1) | 4 | 729.7242 |
| fit28 <- lmer(Juveniles~MicrobiomeType + (1\|Region:Pond:Genotype) , data=dataN1) | 4 | 729.1697 |
| fit29 <- lmer(Juveniles~Region + (1\|Region:Pond:Genotype) , data=dataN1) | 4 | 729.1515 |
| fit30 <- lmer(Juveniles~Pond + (1\|Region:Pond:Genotype) , data=dataN1) | 8 | 701.7124 |
| fit31 <- lmer(Juveniles~Genotype + (1\|Region:Pond:Genotype) , data=dataN1) | 10 | 693.0774 |
| **Model: LMER with random factor (1\|Region:Pond) on total number of juveniles** | **df** | **AIC** |
| fit1 <- lmer(Juveniles~Diet*MicrobiomeType*Region*Pond*Genotype + (1\|Region:Pond) , data=dataN1) | 34 | 567.8499 |
| fit2 <- lmer(Juveniles~Diet*MicrobiomeType*Region*Pond + (1\|Region:Pond) , data=dataN1) | 26 | 607.9774 |
| fit3 <- lmer(Juveniles~Diet*MicrobiomeType*Region*Genotype + (1\|Region:Pond) , data=dataN1) | 34 | 567.8499 |
| fit4 <- lmer(Juveniles~Diet*MicrobiomeType*Pond*Genotype + (1\|Region:Pond) , data=dataN1) | 34 | 565.0774 |
| fit5 <- lmer(Juveniles~Diet*Region*Pond*Genotype + (1\|Region:Pond) , data=dataN1) | 34 | 565.0774 |
| fit6 <- lmer(Juveniles~MicrobiomeType*Region*Pond*Genotype + (1\|Region:Pond) , data=dataN1) | 18 | 660.8931 |
| fit7 <- lmer(Juveniles~Diet*MicrobiomeType*Region + (1\|Region:Pond) , data=dataN1) | 10 | 702.4561 |
| fit8 <- lmer(Juveniles~Diet*MicrobiomeType*Pond + (1\|Region:Pond) , data=dataN1) | 26 | 605.2049 |
| fit9 <- lmer(Juveniles~Diet*Region*Pond + (1\|Region:Pond) , data=dataN1) | 26 | 605.2049 |
| fit10 <- lmer(Juveniles~MicrobiomeType*Region*Pond + (1\|Region:Pond) , data=dataN1) | 14 | 679.4517 |
| fit11<- lmer(Juveniles~Diet*MicrobiomeType*Genotype + (1\|Region:Pond) , data=dataN1) | 34 | 565.0774 |
| fit12<- lmer(Juveniles~Diet*Region*Genotype + (1\|Region:Pond) , data=dataN1) | 34 | 565.0774 |
| fit13<- lmer(Juveniles~MicrobiomeType*Region*Genotype + (1\|Region:Pond) , data=dataN1) | 18 | 660.8931 |
| fit14<- lmer(Juveniles~Diet*Pond*Genotype + (1\|Region:Pond) , data=dataN1) | 18 | 660.7503 |
| fit15<- lmer(Juveniles~MicrobiomeType*Pond*Genotype + (1\|Region:Pond) , data=dataN1) | 18 | 659.5068 |
| fit16<- lmer(Juveniles~Region*Pond*Genotype + (1\|Region:Pond) , data=dataN1) | 18 | 659.5068 |
| fit17 <- lmer(Juveniles~Diet*MicrobiomeType + (1\|Region:Pond) , data=dataN1) | 6 | 721.8446 |
| fit18 <- lmer(Juveniles~Diet*Region + (1\|Region:Pond) , data=dataN1) | 6 | 724.2793 |
| fit19 <- lmer(Juveniles~Diet*Pond + (1\|Region:Pond) , data=dataN1) | 14 | 677.9056 |
| fit20 <- lmer(Juveniles~Diet*Genotype + (1\|Region:Pond) , data=dataN1) | 18 | 660.7503 |
| fit21 <- lmer(Juveniles~MicrobiomeType*Region + (1\|Region:Pond) , data=dataN1) | 6 | 720.2809 |
| fit22 <- lmer(Juveniles~MicrobiomeType*Pond + (1\|Region:Pond) , data=dataN1) | 14 | 678.0654 |
| fit23 <- lmer(Juveniles~MicrobiomeType*Genotype + (1\|Region:Pond) , data=dataN1) | 18 | 659.5068 |
| fit24 <- lmer(Juveniles~Region*Pond + (1\|Region:Pond) , data=dataN1) | 14 | 678.0654 |
| fit25 <- lmer(Juveniles~Region*Genotype + (1\|Region:Pond) , data=dataN1) | 18 | 659.5068 |
| fit26 <- lmer(Juveniles~Pond*Genotype + (1\|Region:Pond) , data=dataN1) | 10 | 693.0774 |
| fit27 <- lmer(Juveniles~Diet + (1\|Region:Pond) , data=dataN1) | 4 | 730.4548 |
| fit28 <- lmer(Juveniles~MicrobiomeType + (1\|Region:Pond) , data=dataN1) | 4 | 729.5013 |
| fit29 <- lmer(Juveniles~Region + (1\|Region:Pond) , data=dataN1) | 4 | 729.4674 |
| fit30 <- lmer(Juveniles~Pond + (1\|Region:Pond) , data=dataN1) | 8 | 701.7124 |
| fit31 <- lmer(Juveniles~Genotype + (1\|Region:Pond) , data=dataN1) | 10 | 693.0774 |
| **Model: GLM on total number of juveniles** | **df** | **AIC** |
| fit1 <- glm(Juveniles~Diet*MicrobiomeType*Region*Pond*Genotype, data=dataN1) | 33 | 728.7506 |
| fit2 <- glm(Juveniles~Diet*MicrobiomeType*Region*Pond, data=dataN1) | 25 | 727.3817 |
| fit3 <- glm(Juveniles~Diet*MicrobiomeType*Region*Genotype data=dataN1) | 33 | 728.7506 |
| fit4 <- glm(Juveniles~Diet*MicrobiomeType*Pond*Genotype, data=dataN1) | 33 | 728.7506 |
| fit5 <- glm(Juveniles~Diet*Region*Pond*Genotype, data=dataN1) | 33 | 728.7506 |
| fit6 <- glm(Juveniles~MicrobiomeType*Region*Pond*Genotype, data=dataN1) | 17 | 733.2143 |
| fit7 <- glm(Juveniles~Diet*MicrobiomeType*Region, data=dataN1) | 9 | 739.1909 |
| fit8 <- glm(Juveniles~Diet*MicrobiomeType*Pond, data=dataN1) | 25 | 727.3817 |
| fit9 <- glm(Juveniles~Diet*Region*Pond, data=dataN1) | 25 | 727.3817 |
| fit10 <- glm(Juveniles~MicrobiomeType*Region*Pond, data=dataN1) | 13 | 732.2617 |
| fit11<- glm(Juveniles~Diet*MicrobiomeType*Genotype, data=dataN1) | 33 | 728.7506 |
| fit12<- glm(Juveniles~Diet*Region*Genotype, data=dataN1) | 33 | 728.7506 |
| fit13<- glm(Juveniles~MicrobiomeType*Region*Genotype, data=dataN1) | 17 | 733.2143 |
| fit14<- glm(Juveniles~Diet*Pond*Genotype, data=dataN1) | 17 | 734.7150 |
| fit15<- glm(Juveniles~MicrobiomeType*Pond*Genotype, data=dataN1) | 17 | 733.2143 |
| fit16<- glm(Juveniles~Region*Pond*Genotype, data=dataN1) | 17 | 733.2143 |
| fit17 <- glm(Juveniles~Diet*MicrobiomeType, data=dataN1) | 5 | 740.5595 |
| fit18 <- glm(Juveniles~Diet*Region, data=dataN1) | 5 | 741.6364 |
| fit19 <- glm(Juveniles~Diet*Pond, data=dataN1) | 13 | 732.0911 |
| fit20 <- glm(Juveniles~Diet*Genotype, data=dataN1) | 17 | 734.7150 |
| fit21 <- glm(Juveniles~MicrobiomeType*Region, data=dataN1) | 5 | 736.2109 |
| fit22 <- glm(Juveniles~MicrobiomeType*Pond, data=dataN1) | 13 | 732.2617 |
| fit23 <- glm(Juveniles~MicrobiomeType*Genotype, data=dataN1) | 17 | 733.2143 |
| fit24 <- glm(Juveniles~Region*Pond, data=dataN1) | 13 | 732.2617 |
| fit25 <- glm(Juveniles~Region*Genotype, data=dataN1) | 17 | 733.2143 |
| fit26 <- glm(Juveniles~Pond*Genotype, data=dataN1) | 9 | 723.4948 |
| fit27 <- glm(Juveniles~Diet, data=dataN1) | 3 | 738.0408 |
| fit28 <- glm(Juveniles~MicrobiomeType, data=dataN1) | 3 | 738.0400 |
| fit29 <- glm(Juveniles~Region, data=dataN1) | 3 | 738.2020 |
| fit30 <- glm(Juveniles~Pond, data=dataN1) | 7 | 723.6271 |
| fit31 <- glm(Juveniles~Genotype, data=dataN1) | 9 | 723.4948 |
| **Model: LMER with random factor (1\|Region:Pond:Genotype) on body size with Time as a factor** | **df** | **AIC** |
| fit1 <- lmer(BodySize~Time*Diet*MicrobiomeType*Region*Pond*Genotype + (1\|Region:Pond:Genotype) , data=dataNb1) | 130 | 3972.796 |
| fit2 <- lmer(BodySize~Time*Diet*MicrobiomeType*Region*Pond + (1\|Region:Pond:Genotype) , data=dataNb1) | 98 | 4346.979 |
| fit3 <- lmer(BodySize~Time*Diet*MicrobiomeType*Region*Genotype + (1\|Region:Pond:Genotype) , data=dataNb1) | 130 | 3972.796 |
| fit4 <- lmer(BodySize~Time*Diet*MicrobiomeType*Pond*Genotype + (1\|Region:Pond:Genotype) , data=dataNb1) | 130 | 3961.706 |
| fit5 <- lmer(BodySize~Time*Diet*Region*Pond*Genotype + (1\|Region:Pond:Genotype) , data=dataNb1) | 130 | 3961.706 |
| fit6 <- lmer(BodySize~Time*MicrobiomeType*Region*Pond*Genotype + (1\|Region:Pond:Genotype) , data=dataNb1) | 66 | 4744.633 |
| fit7 <- lmer(BodySize~Time*Diet*MicrobiomeType*Region + (1\|Region:Pond:Genotype) , data=dataNb1) | 34 | 5120.824 |
| fit8 <- lmer(BodySize~Time*Diet*MicrobiomeType*Pond + (1\|Region:Pond:Genotype) , data=dataNb1) | 98 | 4335.889 |
| fit9 <- lmer(BodySize~Time*Diet*Region*Pond + (1\|Region:Pond:Genotype) , data=dataNb1) | 98 | 4335.889 |
| fit10 <- lmer(BodySize~Time*MicrobiomeType*Region*Pond + (1\|Region:Pond:Genotype) , data=dataNb1) | 50 | 4921.374 |
| fit11<- lmer(BodySize~Time*Diet*MicrobiomeType*Genotype + (1\|Region:Pond:Genotype) , data=dataNb1) | 130 | 3961.706 |
| fit12<- lmer(BodySize~Time*Diet*Region*Genotype + (1\|Region:Pond:Genotype) , data=dataNb1) | 130 | 3961.706 |
| fit13<- lmer(BodySize~Time*MicrobiomeType*Region*Genotype + (1\|Region:Pond:Genotype) , data=dataNb1) | 66 | 4744.633 |
| fit14<- lmer(BodySize~Time*Diet*Pond*Genotype + (1\|Region:Pond:Genotype) , data=dataNb1) | 66 | 4752.047 |
| fit15<- lmer(BodySize~Time*MicrobiomeType*Pond*Genotype + (1\|Region:Pond:Genotype) , data=dataNb1) | 66 | 4739.088 |
| fit16<- lmer(BodySize~Time*Region*Pond*Genotype + (1\|Region:Pond:Genotype) , data=dataNb1) | 66 | 4739.088 |
| fit17 <- lmer(BodySize~Time*Diet*MicrobiomeType + (1\|Region:Pond:Genotype) , data=dataNb1) | 18 | 5320.426 |
| fit18 <- lmer(BodySize~Time*Diet*Region + (1\|Region:Pond:Genotype) , data=dataNb1) | 18 | 5323.846 |
| fit19 <- lmer(BodySize~Time*Diet*Pond + (1\|Region:Pond:Genotype) , data=dataNb1) | 50 | 4932.989 |
| fit20 <- lmer(BodySize~Time*Diet*Genotype + (1\|Region:Pond:Genotype) , data=dataNb1) | 66 | 4752.047 |
| fit21 <- lmer(BodySize~Time*MicrobiomeType*Region + (1\|Region:Pond:Genotype) , data=dataNb1) | 18 | 5302.237 |
| fit22 <- lmer(BodySize~Time*MicrobiomeType*Pond + (1\|Region:Pond:Genotype) , data=dataNb1) | 50 | 4915.829 |
| fit23 <- lmer(BodySize~Time*MicrobiomeType*Genotype + (1\|Region:Pond:Genotype) , data=dataNb1) | 66 | 4739.088 |
| fit24 <- lmer(BodySize~Time*Region*Pond + (1\|Region:Pond:Genotype) , data=dataNb1) | 50 | 4915.829 |
| fit25 <- lmer(BodySize~Time*Region*Genotype + (1\|Region:Pond:Genotype) , data=dataNb1) | 66 | 4739.088 |
| fit26 <- lmer(BodySize~Time*Pond*Genotype + (1\|Region:Pond:Genotype) , data=dataNb1) | 34 | 5116.842 |
| fit27 <- lmer(BodySize~Time*Diet + (1\|Region:Pond:Genotype) , data=dataNb1) | 10 | 5409.607 |
| fit28 <- lmer(BodySize~Time*MicrobiomeType + (1\|Region:Pond:Genotype) , data=dataNb1) | 10 | 5408.399 |
| fit29 <- lmer(BodySize~Time*Region + (1\|Region:Pond:Genotype) , data=dataNb1) | 10 | 5405.053 |
| fit30 <- lmer(BodySize~Time*Pond + (1\|Region:Pond:Genotype) , data=dataNb1) | 26 | 5202.653 |
| fit31 <- lmer(BodySize~Time*Genotype + (1\|Region:Pond:Genotype) , data=dataNb1) | 34 | 5116.842 |
| **Model: LMER with random factor (1\|Region:Pond:Genotype) on body size and without Time as a factor** | **df** | **AIC** |
| fit6 <- lmer(BodySize~MicrobiomeType*Region*Pond*Genotype + (1\|Region:Pond:Genotype) , data=dataNb1) | 34 | 5924.408 |
| fit7 <- lmer(BodySize~Diet*MicrobiomeType*Region + (1\|Region:Pond:Genotype) , data=dataNb1) | 10 | 6213.966 |
| fit8 <- lmer(BodySize~ Diet*MicrobiomeType*Pond + (1\|Region:Pond:Genotype) , data=dataNb1) | 26 | 6017.975 |
| fit9 <- lmer(BodySize~ Diet*Region*Pond + (1\|Region:Pond:Genotype) , data=dataNb1) | 26 | 6017.975 |
| fit10 <- lmer(BodySize~ MicrobiomeType*Region*Pond + (1\|Region:Pond:Genotype) , data=dataNb1) | 14 | 6165.752 |
| fit11<- lmer(BodySize~ Diet*MicrobiomeType*Genotype + (1\|Region:Pond:Genotype) , data=dataNb1) | 34 | 5921.636 |
| fit12<- lmer(BodySize~ Diet*Region*Genotype + (1\|Region:Pond:Genotype) , data=dataNb1) | 34 | 5921.636 |
| fit13<- lmer(BodySize~ MicrobiomeType*Region*Genotype + (1\|Region:Pond:Genotype) , data=dataNb1) | 18 | 6120.107 |
| fit14<- lmer(BodySize~ Diet*Pond*Genotype + (1\|Region:Pond:Genotype) , data=dataNb1) | 18 | 6119.857 |
| fit15<- lmer(BodySize~ MicrobiomeType*Pond*Genotype + (1\|Region:Pond:Genotype) , data=dataNb1) | 18 | 6118.721 |
| fit16<- lmer(BodySize~ Region*Pond*Genotype + (1\|Region:Pond:Genotype) , data=dataNb1) | 18 | 6118.721 |
| fit17 <- lmer(BodySize~ Diet*MicrobiomeType + (1\|Region:Pond:Genotype) , data=dataNb1) | 6 | 6260.937 |
| fit18 <- lmer(BodySize~ Diet*Region + (1\|Region:Pond:Genotype) , data=dataNb1) | 6 | 6261.900 |
| fit19 <- lmer(BodySize~ Diet*Pond + (1\|Region:Pond:Genotype) , data=dataNb1) | 14 | 6165.301 |
| fit20 <- lmer(BodySize~Diet*Genotype + (1\|Region:Pond:Genotype) , data=dataNb1) | 18 | 6119.857 |
| fit21 <- lmer(BodySize~ MicrobiomeType*Region + (1\|Region:Pond:Genotype) , data=dataNb1) | 6 | 6257.900 |
| fit22 <- lmer(BodySize~ MicrobiomeType*Pond + (1\|Region:Pond:Genotype) , data=dataNb1) | 14 | 6164.366 |
| fit23 <- lmer(BodySize~ MicrobiomeType*Genotype + (1\|Region:Pond:Genotype) , data=dataNb1) | 18 | 6118.721 |
| fit24 <- lmer(BodySize~ Region*Pond + (1\|Region:Pond:Genotype) , data=dataNb1) | 14 | 6164.366 |
| fit25 <- lmer(BodySize~ Region*Genotype + (1\|Region:Pond:Genotype) , data=dataNb1) | 18 | 6118.721 |
| fit26 <- lmer(BodySize~ Pond*Genotype + (1\|Region:Pond:Genotype) , data=dataNb1) | 10 | 6211.750 |
| fit27 <- lmer(BodySize~ Diet + (1\|Region:Pond:Genotype) , data=dataNb1) | 4 | 6281.985 |
| fit28 <- lmer(BodySize~MicrobiomeType + (1\|Region:Pond:Genotype) , data=dataNb1) | 4 | 6282.222 |
| fit29 <- lmer(BodySize~ Region + (1\|Region:Pond:Genotype) , data=dataNb1) | 4 | 6282.270 |
| fit30 <- lmer(BodySize~ Pond + (1\|Region:Pond:Genotype) , data=dataNb1) | 8 | 6233.603 |
| fit31 <- lmer(BodySize~ Genotype + (1\|Region:Pond:Genotype) , data=dataNb1) | 10 | 6211.750 |
| **Model: LMER with random factor 1\|Region:Pond) on body size with time as a factor** | **df** | **AIC** |
| fit1 <- lmer(BodySize~Time*Diet*MicrobiomeType*Region*Pond*Genotype + (1\|Region:Pond) , data=dataNb1) | 130 | 3972.796 |
| fit2 <- lmer(BodySize~Time*Diet*MicrobiomeType*Region*Pond + (1\|Region:Pond) , data=dataNb1) | 98 | 4354.474 |
| fit3 <- lmer(BodySize~Time*Diet*MicrobiomeType*Region*Genotype + (1\|Region:Pond) , data=dataNb1) | 130 | 3972.796 |
| fit4 <- lmer(BodySize~Time*Diet*MicrobiomeType*Pond*Genotype + (1\|Region:Pond) , data=dataNb1) | 130 | 3961.706 |
| fit5 <- lmer(BodySize~Time*Diet*Region*Pond*Genotype + (1\|Region:Pond) , data=dataNb1) | 130 | 3961.706 |
| fit6 <- lmer(BodySize~Time*MicrobiomeType*Region*Pond*Genotype + (1\|Region:Pond) , data=dataNb1) | 66 | 4744.633 |
| fit7 <- lmer(BodySize~Time*Diet*MicrobiomeType*Region + (1\|Region:Pond) , data=dataNb1) | 34 | 5127.532 |
| fit8 <- lmer(BodySize~Time*Diet*MicrobiomeType*Pond + (1\|Region:Pond) , data=dataNb1) | 98 | 4343.383 |
| fit9 <- lmer(BodySize~Time*Diet*Region*Pond + (1\|Region:Pond) , data=dataNb1) | 98 | 4343.383 |
| fit10 <- lmer(BodySize~Time*MicrobiomeType*Region*Pond + (1\|Region:Pond) , data=dataNb1) | 50 | 4929.329 |
| fit11<- lmer(BodySize~Time*Diet*MicrobiomeType*Genotype + (1\|Region:Pond) , data=dataNb1) | 130 | 3961.706 |
| fit12<- lmer(BodySize~Time*Diet*Region*Genotype + (1\|Region:Pond) , data=dataNb1) | 130 | 3961.706 |
| fit13<- lmer(BodySize~Time*MicrobiomeType*Region*Genotype + (1\|Region:Pond) , data=dataNb1) | 66 | 4744.633 |
| fit14<- lmer(BodySize~Time*Diet*Pond*Genotype + (1\|Region:Pond) , data=dataNb1) | 66 | 4753.938 |
| fit15<- lmer(BodySize~Time*MicrobiomeType*Pond*Genotype + (1\|Region:Pond) , data=dataNb1) | 66 | 4739.088 |
| fit16<- lmer(BodySize~Time*Region*Pond*Genotype + (1\|Region:Pond) , data=dataNb1) | 66 | 4739.088 |
| fit17 <- lmer(BodySize~Time*Diet*MicrobiomeType + (1\|Region:Pond) , data=dataNb1) | 18 | 5325.497 |
| fit18 <- lmer(BodySize~Time*Diet*Region + (1\|Region:Pond) , data=dataNb1) | 18 | 5325.805 |
| fit19 <- lmer(BodySize~Time*Diet*Pond + (1\|Region:Pond) , data=dataNb1) | 50 | 4940.432 |
| fit20 <- lmer(BodySize~Time*Diet*Genotype + (1\|Region:Pond) , data=dataNb1) | 66 | 4753.938 |
| fit21 <- lmer(BodySize~Time*MicrobiomeType*Region + (1\|Region:Pond) , data=dataNb1) | 18 | 5309.176 |
| fit22 <- lmer(BodySize~Time*MicrobiomeType*Pond + (1\|Region:Pond) , data=dataNb1) | 50 | 4923.783 |
| fit23 <- lmer(BodySize~Time*MicrobiomeType*Genotype + (1\|Region:Pond) , data=dataNb1) | 66 | 4739.088 |
| fit24 <- lmer(BodySize~Time*Region*Pond + (1\|Region:Pond) , data=dataNb1) | 50 | 4923.783 |
| fit25 <- lmer(BodySize~Time*Region*Genotype + (1\|Region:Pond) , data=dataNb1) | 66 | 4739.088 |
| fit26 <- lmer(BodySize~Time*Pond*Genotype + (1\|Region:Pond) , data=dataNb1) | 34 | 5118.490 |
| fit27 <- lmer(BodySize~Time*Diet + (1\|Region:Pond) , data=dataNb1) | 10 | 5412.761 |
| fit28 <- lmer(BodySize~Time*MicrobiomeType + (1\|Region:Pond) , data=dataNb1) | 10 | 5412.071 |
| fit29 <- lmer(BodySize~Time*Region + (1\|Region:Pond) , data=dataNb1) | 10 | 5408.810 |
| fit30 <- lmer(BodySize~Time*Pond + (1\|Region:Pond) , data=dataNb1) | 26 | 5211.151 |
| fit31 <- lmer(BodySize~Time*Genotype + (1\|Region:Pond) , data=dataNb1) | 34 | 5118.490 |
| **Model: LMER with random factor (1\|Region:Pond) on body size without Time as a factor** | **df** | **AIC** |
| fit6 <- lmer(BodySize~MicrobiomeType*Region*Pond*Genotype + (1\|Region:Pond) , data=dataNb1) | 34 | 5924.408 |
| fit7 <- lmer(BodySize~Diet*MicrobiomeType*Region + (1\|Region:Pond) , data=dataNb1) | 10 | 6213.966 |
| fit8 <- lmer(BodySize~ Diet*MicrobiomeType*Pond + (1\|Region:Pond) , data=dataNb1) | 26 | 6017.975 |
| fit9 <- lmer(BodySize~ Diet*Region*Pond + (1\|Region:Pond) , data=dataNb1) | 26 | 6017.975 |
| fit10 <- lmer(BodySize~ MicrobiomeType*Region*Pond + (1\|Region:Pond) , data=dataNb1) | 14 | 6165.752 |
| fit11<- lmer(BodySize~ Diet*MicrobiomeType*Genotype + (1\|Region:Pond) , data=dataNb1) | 34 | 5921.636 |
| fit12<- lmer(BodySize~ Diet*Region*Genotype + (1\|Region:Pond) , data=dataNb1) | 34 | 5921.636 |
| fit13<- lmer(BodySize~ MicrobiomeType*Region*Genotype + (1\|Region:Pond) , data=dataNb1) | 18 | 6120.107 |
| fit14<- lmer(BodySize~ Diet*Pond*Genotype + (1\|Region:Pond) , data=dataNb1) | 18 | 6119.857 |
| fit15<- lmer(BodySize~ MicrobiomeType*Pond*Genotype + (1\|Region:Pond) , data=dataNb1) | 18 | 6118.721 |
| fit16<- lmer(BodySize~ Region*Pond*Genotype + (1\|Region:Pond) , data=dataNb1) | 18 | 6118.721 |
| fit17 <- lmer(BodySize~ Diet*MicrobiomeType + (1\|Region:Pond) , data=dataNb1) | 6 | 6260.781 |
| fit18 <- lmer(BodySize~ Diet*Region + (1\|Region:Pond) , data=dataNb1) | 6 | 6261.460 |
| fit19 <- lmer(BodySize~ Diet*Pond + (1\|Region:Pond) , data=dataNb1) | 14 | 6165.301 |
| fit20 <- lmer(BodySize~Diet*Genotype + (1\|Region:Pond) , data=dataNb1) | 18 | 6119.857 |
| fit21 <- lmer(BodySize~ MicrobiomeType*Region + (1\|Region:Pond) , data=dataNb1) | 6 | 6257.900 |
| fit22 <- lmer(BodySize~ MicrobiomeType*Pond + (1\|Region:Pond) , data=dataNb1) | 14 | 6164.366 |
| fit23 <- lmer(BodySize~ MicrobiomeType*Genotype + (1\|Region:Pond) , data=dataNb1) | 18 | 6118.721 |
| fit24 <- lmer(BodySize~ Region*Pond + (1\|Region:Pond) , data=dataNb1) | 14 | 6164.366 |
| fit25 <- lmer(BodySize~ Region*Genotype + (1\|Region:Pond) , data=dataNb1) | 18 | 6118.721 |
| fit26 <- lmer(BodySize~ Pond*Genotype + (1\|Region:Pond) , data=dataNb1) | 10 | 6211.750 |
| fit27 <- lmer(BodySize~ Diet + (1\|Region:Pond) , data=dataNb1) | 4 | 6281.826 |
| fit28 <- lmer(BodySize~MicrobiomeType + (1\|Region:Pond) , data=dataNb1) | 4 | 6281.852 |
| fit29 <- lmer(BodySize~ Region + (1\|Region:Pond) , data=dataNb1) | 4 | 6281.857 |
| fit30 <- lmer(BodySize~ Pond + (1\|Region:Pond) , data=dataNb1) | 8 | 6233.603 |
| fit31 <- lmer(BodySize~ Genotype + (1\|Region:Pond) , data=dataNb1) | 10 | 6211.750 |
| **Model: GLM on body size with Time as a factor** | **df** | **AIC** |
| fit1 <- glm(BodySize~Time*Diet*MicrobiomeType*Region*Pond*Genotype, data=dataNb1) | 129 | 5524.048 |
| fit2 <- glm(BodySize~Time*Diet*MicrobiomeType*Region*Pond, data=dataNb1) | 97 | 5511.991 |
| fit3 <- glm(BodySize~Time*Diet*MicrobiomeType*Region*Genotype data=dataNb1) | 129 | 5524.048 |
| fit4 <- glm(BodySize~Time*Diet*MicrobiomeType*Pond*Genotype, data=dataNb1) | 129 | 5524.048 |
| fit5 <- glm(BodySize~Time*Diet*Region*Pond*Genotype, data=dataNb1) | 129 | 5524.048 |
| fit6 <- glm(BodySize~Time*MicrobiomeType*Region*Pond*Genotype, data=dataNb1) | 65 | 5480.127 |
| fit7 <- glm(BodySize~Time*Diet*MicrobiomeType*Region, data=dataNb1) | 33 | 5493.075 |
| fit8 <- glm(BodySize~Time*Diet*MicrobiomeType*Pond, data=dataNb1) | 97 | 5511.991 |
| fit9 <- glm(BodySize~Time*Diet*Region*Pond, data=dataNb1) | 97 | 5511.991 |
| fit10 <- glm(BodySize~Time*MicrobiomeType*Region*Pond, data=dataNb1) | 49 | 5476.578 |
| fit11<- glm(BodySize~Time*Diet*MicrobiomeType*Genotype, data=dataNb1) | 129 | 5524.048 |
| fit12<- glm(BodySize~Time*Diet*Region*Genotype, data=dataNb1) | 129 | 5524.048 |
| fit13<- glm(BodySize~Time*MicrobiomeType*Region*Genotype, data=dataNb1) | 65 | 5480.127 |
| fit14<- glm(BodySize~Time*Diet*Pond*Genotype, data=dataNb1) | 65 | 5500.340 |
| fit15<- glm(BodySize~Time*MicrobiomeType*Pond*Genotype, data=dataNb1) | 65 | 5480.127 |
| fit16<- glm(BodySize~Time*Region*Pond*Genotype, data=dataNb1) | 65 | 5480.127 |
| fit17 <- glm(BodySize~Time*Diet*MicrobiomeType, data=dataNb1) | 17 | 5530.888 |
| fit18 <- glm(BodySize~Time*Diet*Region, data=dataNb1) | 17 | 5539.132 |
| fit19 <- glm(BodySize~Time*Diet*Pond, data=dataNb1) | 49 | 8597.551 |
| fit20 <- glm(BodySize~Time*Diet*Genotype, data=dataNb1) | 65 | 5500.340 |
| fit21 <- glm(BodySize~Time*MicrobiomeType*Region, data=dataNb1) | 17 | 5488.729 |
| fit22 <- glm(BodySize~Time*MicrobiomeType*Pond, data=dataNb1) | 49 | 5476.578 |
| fit23 <- glm(BodySize~Time*MicrobiomeType*Genotype, data=dataNb1) | 65 | 5480.127 |
| fit24 <- glm(BodySize~Time*Region*Pond, data=dataNb1) | 49 | 5476.578 |
| fit25 <- glm(BodySize~Time*Region*Genotype, data=dataNb1) | 65 | 5480.127 |
| fit26 <- glm(BodySize~Time*Pond*Genotype, data=dataNb1) | 33 | 5471.863 |
| fit27 <- glm(BodySize~Time*Diet, data=dataNb1) | 9 | 5532.384 |
| fit28 <- glm(BodySize~Time*MicrobiomeType, data=dataNb1) | 9 | 5533.526 |
| fit29 <- glm(BodySize~Time*Region, data=dataNb1) | 9 | 5531.236 |
| fit30 <- glm(BodySize~Time*Pond, data=dataNb1) | 25 | 5474.772 |
| fit31 <- glm(BodySize~Time*Genotype, data=dataNb1) | 33 | 5471.863 |
| **Model: GLM on body size without Time as a factor.** | **df** | **AIC** |
| fit6 <- glm(BodySize~ MicrobiomeType*Region*Pond*Genotype, data=dataNb1) | 33 | 6348.031 |
| fit7 <- glm(BodySize~ Diet*MicrobiomeType*Region, data=dataNb1) | 9 | 6307.237 |
| fit8 <- glm(BodySize~ Diet*MicrobiomeType*Pond, data=dataNb1) | 25 | 6334.464 |
| fit9 <- glm(BodySize~ Diet*Region*Pond, data=dataNb1) | 25 | 6334.464 |
| fit10 <- glm(BodySize~ MicrobiomeType*Region*Pond, data=dataNb1) | 13 | 6312.808 |
| fit11<- glm(BodySize~ Diet*MicrobiomeType*Genotype, data=dataNb1) | 33 | 6348.031 |
| fit12<- glm(BodySize~ Diet*Region*Genotype, data=dataNb1) | 33 | 6348.031 |
| fit13<- glm(BodySize~ MicrobiomeType*Region*Genotype, data=dataNb1) | 17 | 6318.980 |
| fit14<- glm(BodySize~ Diet*Pond*Genotype, data=dataNb1) | 17 | 6320.171 |
| fit15<- glm(BodySize~ MicrobiomeType*Pond*Genotype, data=dataNb1) | 17 | 6318.980 |
| fit16<- glm(BodySize~ Region*Pond*Genotype, data=dataNb1) | 17 | 6318.980 |
| fit17 <- glm(BodySize~ Diet*MicrobiomeType, data=dataNb1) | 5 | 6303.755 |
| fit18 <- glm(BodySize~ Diet*Region, data=dataNb1) | 5 | 6304.763 |
| fit19 <- glm(BodySize~ Diet*Pond, data=dataNb1) | 13 | 6312.808 |
| fit20 <- glm(BodySize~ Diet*Genotype, data=dataNb1) | 17 | 6320.171 |
| fit21 <- glm(BodySize~ MicrobiomeType*Region, data=dataNb1) | 5 | 6300.707 |
| fit22 <- glm(BodySize~ MicrobiomeType*Pond, data=dataNb1) | 13 | 6312.808 |
| fit23 <- glm(BodySize~ MicrobiomeType*Genotype, data=dataNb1) | 17 | 6318.980 |
| fit24 <- glm(BodySize~ Region*Pond, data=dataNb1) | 13 | 6312.808 |
| fit25 <- glm(BodySize~ Region*Genotype, data=dataNb1) | 17 | 6318.980 |
| fit26 <- glm(BodySize~ Pond*Genotype, data=dataNb1) | 9 | 6305.247 |
| fit27 <- glm(BodySize~ Diet, data=dataNb1) | 3 | 6301.001 |
| fit28 <- glm(BodySize~ MicrobiomeType, data=dataNb1) | 3 | 6301.240 |
| fit29 <- glm(BodySize~ Region, data=dataNb1) | 3 | 6301.290 |
| fit30 <- glm(BodySize~ Pond, data=dataNb1) | 7 | 6302.609 |
| fit31 <- glm(BodySize~ Genotype, data=dataNb1) | 9 | 6305.247 |
| **Model: LMER with random factor (1\|Region:Pond:Genotype) on Shannon entropy** | **df** | **AIC** |
| fit1 <- lmer(ShannonEntropy~Diet*MicrobiomeType*Region*Pond*Genotype + (1\|Region:Pond:Genotype) , data=div_samp) | 34 | 83.59273 |
| fit2 <- lmer(ShannonEntropy~Diet*MicrobiomeType*Region*Pond + (1\|Region:Pond:Genotype) , data=div_samp) | 26 | 148.20455 |
| fit3 <- lmer(ShannonEntropy~Diet*MicrobiomeType*Region*Genotype + (1\|Region:Pond:Genotype) , data=div_samp) | 34 | 83.59273 |
| fit4 <- lmer(ShannonEntropy~Diet*MicrobiomeType*Pond*Genotype + (1\|Region:Pond:Genotype) , data=div_samp) | 34 | 83.59273 |
| fit5 <- lmer(ShannonEntropy~Diet*Region*Pond*Genotype + (1\|Region:Pond:Genotype) , data=div_samp) | 21 | 188.63398 |
| fit6 <- lmer(ShannonEntropy~MicrobiomeType*Region*Pond*Genotype + (1\|Region:Pond:Genotype) , data=div_samp) | 19 | 193.40674 |
| fit7 <- lmer(ShannonEntropy~Diet*MicrobiomeType*Region + (1\|Region:Pond:Genotype) , data=div_samp) | 14 | 228.37080 |
| fit8 <- lmer(ShannonEntropy~Diet*MicrobiomeType*Pond + (1\|Region:Pond:Genotype) , data=div_samp) | 26 | 148.20455 |
| fit9 <- lmer(ShannonEntropy~Diet*Region*Pond + (1\|Region:Pond:Genotype) , data=div_samp) | 17 | 211.44541 |
| fit10 <- lmer(ShannonEntropy~MicrobiomeType*Region*Pond + (1\|Region:Pond:Genotype) , data=div_samp) | 15 | 215.89417 |
| fit11<- lmer(ShannonEntropy~Diet*MicrobiomeType*Genotype + (1\|Region:Pond:Genotype) , data=div_samp) | 34 | 83.59273 |
| fit12<- lmer(ShannonEntropy~Diet*Region*Genotype + (1\|Region:Pond:Genotype) , data=div_samp) | 21 | 188.63398 |
| fit13<- lmer(ShannonEntropy~MicrobiomeType*Region*Genotype + (1\|Region:Pond:Genotype) , data=div_samp) | 19 | 192.40674 |
| fit14<- lmer(ShannonEntropy~Diet*Pond*Genotype + (1\|Region:Pond:Genotype) , data=div_samp) | 21 | 188.63398 |
| fit15<- lmer(ShannonEntropy~MicrobiomeType*Pond*Genotype + (1\|Region:Pond:Genotype) , data=div_samp) | 19 | 192.40674 |
| fit16<- lmer(ShannonEntropy~Region*Pond*Genotype + (1\|Region:Pond:Genotype) , data=div_samp) | 12 | 238.98951 |
| fit17 <- lmer(ShannonEntropy~Diet*MicrobiomeType + (1\|Region:Pond:Genotype) , data=div_samp) | 8 | 260.19644 |
| fit18 <- lmer(ShannonEntropy~Diet*Region + (1\|Region:Pond:Genotype) , data=div_samp) | 6 | 281.78455 |
| fit19 <- lmer(ShannonEntropy~Diet*Pond + (1\|Region:Pond:Genotype) , data=div_samp) | 17 | 211.44541 |
| fit20 <- lmer(ShannonEntropy~Diet*Genotype + (1\|Region:Pond:Genotype) , data=div_samp) | 21 | 188.63398 |
| fit21 <- lmer(ShannonEntropy~MicrobiomeType*Region + (1\|Region:Pond:Genotype) , data=div_samp) | 8 | 258.44870 |
| fit22 <- lmer(ShannonEntropy~MicrobiomeType*Pond + (1\|Region:Pond:Genotype) , data=div_samp) | 15 | 215.89417 |
| fit23 <- lmer(ShannonEntropy~MicrobiomeType*Genotype + (1\|Region:Pond:Genotype) , data=div_samp) | 19 | 192.40674 |
| fit24 <- lmer(ShannonEntropy~Region*Pond + (1\|Region:Pond:Genotype) , data=div_samp) | 10 | 247.65894 |
| fit25 <- lmer(ShannonEntropy~Region*Genotype + (1\|Region:Pond:Genotype) , data=div_samp) | 12 | 238.98951 |
| fit26 <- lmer(ShannonEntropy~Pond*Genotype + (1\|Region:Pond:Genotype) , data=div_samp) | 12 | 238.98951 |
| fit27 <- lmer(ShannonEntropy~Diet + (1\|Region:Pond:Genotype) , data=div_samp) | 4 | 290.42593 |
| fit28 <- lmer(ShannonEntropy~MicrobiomeType + (1\|Region:Pond:Genotype) , data=div_samp) | 5 | 271.52212 |
| fit29 <- lmer(ShannonEntropy~Region + (1\|Region:Pond:Genotype) , data=div_samp) | 4 | 289.10296 |
| fit30 <- lmer(ShannonEntropy~Pond + (1\|Region:Pond:Genotype) , data=div_samp) | 10 | 247.65894 |
| fit31 <- lmer(ShannonEntropy~Genotype + (1\|Region:Pond:Genotype) , data=div_samp) | 12 | 238.98951 |
| **Model: LMER with random factor (1\|Region: Pond) on Shannon entropy** | **df** | **AIC** |
| fit1 <- lmer(ShannonEntropy~Diet*MicrobiomeType*Region*Pond*Genotype + (1\|Region:Pond) , data=div_samp) | 34 | 83.59273 |
| fit2 <- lmer(ShannonEntropy~Diet*MicrobiomeType*Region*Pond + (1\|Region:Pond) , data=div_samp) | 26 | 148.20455 |
| fit3 <- lmer(ShannonEntropy~Diet*MicrobiomeType*Region*Genotype + (1\|Region:Pond) , data=div_samp) | 34 | 83.59273 |
| fit4 <- lmer(ShannonEntropy~Diet*MicrobiomeType*Pond*Genotype + (1\|Region:Pond) , data=div_samp) | 34 | 83.59273 |
| fit5 <- lmer(ShannonEntropy~Diet*Region*Pond*Genotype + (1\|Region:Pond) , data=div_samp) | 21 | 188.63398 |
| fit6 <- lmer(ShannonEntropy~MicrobiomeType*Region*Pond*Genotype + (1\|Region:Pond) , data=div_samp) | 19 | 192.40674 |
| fit7 <- lmer(ShannonEntropy~Diet*MicrobiomeType*Region + (1\|Region:Pond) , data=div_samp) | 14 | 228.37080 |
| fit8 <- lmer(ShannonEntropy~Diet*MicrobiomeType*Pond + (1\|Region:Pond) , data=div_samp) | 26 | 148.20455 |
| fit9 <- lmer(ShannonEntropy~Diet*Region*Pond + (1\|Region:Pond) , data=div_samp) | 17 | 211.44541 |
| fit10 <- lmer(ShannonEntropy~MicrobiomeType*Region*Pond + (1\|Region:Pond) , data=div_samp) | 15 | 215.89417 |
| fit11<- lmer(ShannonEntropy~Diet*MicrobiomeType*Genotype + (1\|Region:Pond) , data=div_samp) | 34 | 83.59273 |
| fit12<- lmer(ShannonEntropy~Diet*Region*Genotype + (1\|Region:Pond) , data=div_samp) | 21 | 188.63398 |
| fit13<- lmer(ShannonEntropy~MicrobiomeType*Region*Genotype + (1\|Region:Pond) , data=div_samp) | 19 | 192.40674 |
| fit14<- lmer(ShannonEntropy~Diet*Pond*Genotype + (1\|Region:Pond) , data=div_samp) | 21 | 188.63398 |
| fit15<- lmer(ShannonEntropy~MicrobiomeType*Pond*Genotype + (1\|Region:Pond) , data=div_samp) | 19 | 192.40674 |
| fit16<- lmer(ShannonEntropy~Region*Pond*Genotype + (1\|Region:Pond) , data=div_samp) | 12 | 238.98951 |
| fit17 <- lmer(ShannonEntropy~Diet*MicrobiomeType + (1\|Region:Pond) , data=div_samp) | 8 | 260.19644 |
| fit18 <- lmer(ShannonEntropy~Diet*Region + (1\|Region:Pond) , data=div_samp) | 6 | 279.72577 |
| fit19 <- lmer(ShannonEntropy~Diet*Pond + (1\|Region:Pond) , data=div_samp) | 17 | 211.44541 |
| fit20 <- lmer(ShannonEntropy~Diet*Genotype + (1\|Region:Pond) , data=div_samp) | 21 | 188.63398 |
| fit21 <- lmer(ShannonEntropy~MicrobiomeType*Region + (1\|Region:Pond) , data=div_samp) | 8 | 258.44870 |
| fit22 <- lmer(ShannonEntropy~MicrobiomeType*Pond + (1\|Region:Pond) , data=div_samp) | 15 | 215.89417 |
| fit23 <- lmer(ShannonEntropy~MicrobiomeType*Genotype + (1\|Region:Pond) , data=div_samp) | 19 | 192.40674 |
| fit24 <- lmer(ShannonEntropy~Region*Pond + (1\|Region:Pond) , data=div_samp) | 10 | 247.65894 |
| fit25 <- lmer(ShannonEntropy~Region*Genotype + (1\|Region:Pond) , data=div_samp) | 12 | 238.98951 |
| fit26 <- lmer(ShannonEntropy~Pond*Genotype + (1\|Region:Pond) , data=div_samp) | 12 | 238.98951 |
| fit27 <- lmer(ShannonEntropy~Diet + (1\|Region:Pond) , data=div_samp) | 4 | 289.02175 |
| fit28 <- lmer(ShannonEntropy~MicrobiomeType + (1\|Region:Pond) , data=div_samp) | 5 | 271.50475 |
| fit29 <- lmer(ShannonEntropy~Region + (1\|Region:Pond) , data=div_samp) | 4 | 287.09977 |
| fit30 <- lmer(ShannonEntropy~Pond + (1\|Region:Pond) , data=div_samp) | 10 | 247.65894 |
| fit31 <- lmer(ShannonEntropy~Genotype + (1\|Region:Pond) , data=div_samp) | 12 | 238.98951 |
| **Model: GLM Shannon entroyp** | **df** | **AIC** |
| fit1 <- glm(ShannonEntropy~Diet*MicrobiomeType*Region*Pond*Genotype, data=div_samp) | 33 | 104.8703 |
| fit2 <- glm(ShannonEntropy~Diet*MicrobiomeType*Region*Pond, data=div_samp) | 25 | 278.4255 |
| fit3 <- glm(ShannonEntropy~Diet*MicrobiomeType*Region*Genotype data=div_samp) | 33 | 104.8703 |
| fit4 <- glm(ShannonEntropy~Diet*MicrobiomeType*Pond*Genotype, data=div_samp) | 33 | 104.8703 |
| fit5 <- glm(ShannonEntropy~Diet*Region*Pond*Genotype, data=div_samp) | 20 | 299.1639 |
| fit6 <- glm(ShannonEntropy~MicrobiomeType*Region*Pond*Genotype, data=div_samp) | 18 | 283.3489 |
| fit7 <- glm(ShannonEntropy~Diet*MicrobiomeType*Region, data=div_samp) | 13 | 293.3227 |
| fit8 <- glm(ShannonEntropy~Diet*MicrobiomeType*Pond, data=div_samp) | 25 | 278.4255 |
| fit9 <- glm(ShannonEntropy~Diet*Region*Pond, data=div_samp) | 16 | 298.0675 |
| fit10 <- glm(ShannonEntropy~MicrobiomeType*Region*Pond, data=div_samp) | 14 | 285.3937 |
| fit11<- glm(ShannonEntropy~Diet*MicrobiomeType*Genotype, data=div_samp) | 33 | 104.8703 |
| fit12<- glm(ShannonEntropy~Diet*Region*Genotype, data=div_samp) | 20 | 299.1639 |
| fit13<- glm(ShannonEntropy~MicrobiomeType*Region*Genotype, data=div_samp) | 18 | 283.3489 |
| fit14<- glm(ShannonEntropy~Diet*Pond*Genotype, data=div_samp) | 20 | 299.1639 |
| fit15<- glm(ShannonEntropy~MicrobiomeType*Pond*Genotype, data=div_samp) | 18 | 283.3489 |
| fit16<- glm(ShannonEntropy~Region*Pond*Genotype, data=div_samp) | 11 | 291.1749 |
| fit17 <- glm(ShannonEntropy~Diet*MicrobiomeType, data=div_samp) | 7 | 287.8047 |
| fit18 <- glm(ShannonEntropy~Diet*Region, data=div_samp) | 5 | 305.5938 |
| fit19 <- glm(ShannonEntropy~Diet*Pond, data=div_samp) | 16 | 298.0675 |
| fit20 <- glm(ShannonEntropy~Diet*Genotype, data=div_samp) | 20 | 299.1639 |
| fit21 <- glm(ShannonEntropy~MicrobiomeType*Region, data=div_samp) | 7 | 285.3937 |
| fit22 <- glm(ShannonEntropy~MicrobiomeType*Pond, data=div_samp) | 14 | 285.3937 |
| fit23 <- glm(ShannonEntropy~MicrobiomeType*Genotype, data=div_samp) | 18 | 283.3489 |
| fit24 <- glm(ShannonEntropy~Region*Pond, data=div_samp) | 9 | 288.2207 |
| fit25 <- glm(ShannonEntropy~Region*Genotype, data=div_samp) | 11 | 291.1749 |
| fit26 <- glm(ShannonEntropy~Pond*Genotype, data=div_samp) | 11 | 291.1749 |
| fit27 <- glm(ShannonEntropy~Diet, data=div_samp) | 3 | 301.6665 |
| fit28 <- glm(ShannonEntropy~MicrobiomeType, data=div_samp) | 4 | 282.1380 |
| fit29 <- glm(ShannonEntropy~Region, data=div_samp) | 3 | 301.7083 |
| fit30 <- glm(ShannonEntropy~Pond, data=div_samp) | 9 | 288.2207 |
| fit31 <- glm(ShannonEntropy~Genotype, data=div_samp) | 11 | 291.1749 |
