# Supplementary material for: Locally adapted gut microbiomes mediate host stress tolerance
Source: ISME J. 2021 Mar 3;15(8):2401–14. doi: 10.1038/s41396-021-00940-y (PMC8319338; doi:10.1038/s41396-021-00940-y)
Supplement: Supplementary file 4 — Table SI4 [file 41396_2021_940_MOESM4_ESM.docx]

Table SI4

|  | *X²* | df | *p*-value |
| --- | --- | --- | --- |
| Diet | 3.9 | 1 | 0.05 |
| Microbiome type | 0.3 | 1 | 0.6 |
| Genotype | 10.1 | 7 | 0.2 |
| Diet x Microbiome type | 4.8 | 3 | 0.2 |
| Diet x Genotype | 22 | 13 | 0.06 (*) |
| Microbiome type x Genotype | 36.2 | 12 | <0.0001*** |
| Diet x Microbiome type x Genotype | 57.9 | 23 | <0.0001*** |
