# Supplementary material for: Locally adapted gut microbiomes mediate host stress tolerance
Source: ISME J. 2021 Mar 3;15(8):2401–14. doi: 10.1038/s41396-021-00940-y (PMC8319338; doi:10.1038/s41396-021-00940-y)
Supplement: Supplementary file 5 — Table SI5 [file 41396_2021_940_MOESM5_ESM.docx]

Table SI5

|  | *F* | df | df.res | *p*-value |
| --- | --- | --- | --- | --- |
| Diet | 0.0260 | 1 | 56.00 | 0.8724 |
| Microbiome type | 0.2500 | 1 | 707.15 | 0.6173 |
| Genotype | 1.1931 | 8 | 665.20 | 0.3004 |
| Diet x Microbiome type | 0.8942 | 1 | 56.00 | 0.3484 |
| Diet x Genotype | 0.3105 | 8 | 56.00 | 0.9590 |
| Microbiome type x Genotype | 0.0788 | 8 | 650.09 | 0.9997 |
| Diet x Microbiome type x Genotype | 0.9691 | 8 | 56.00 | 0.4694 |
