# Supplementary material for: Locally adapted gut microbiomes mediate host stress tolerance
Source: ISME J. 2021 Mar 3;15(8):2401–14. doi: 10.1038/s41396-021-00940-y (PMC8319338; doi:10.1038/s41396-021-00940-y)
Supplement: Supplementary file 6 — Table SI6 [file 41396_2021_940_MOESM6_ESM.docx]

Table SI6

|  | *F* | df | df.res | *p*-value |
| --- | --- | --- | --- | --- |
| Diet | 0.5943 | 1 | 63 | 0.44366 |
| Microbiome type | 0.0156 | 1 | 12393 | 0.90071 |
| Genotype | 0.3281 | 7 | 12912 | 0.94159 |
| Diet x Microbiome type | 3.7680 | 1 | 63 | 0.05672 · |
| Diet x Genotype | 0.9521 | 7 | 63 | 0.47368 |
| Microbiome type x Genotype | 0.0979 | 7 | 11748 | 0.99845 |
| Diet x Microbiome type x Genotype | 2.5663 | 7 | 63 | 0.02161 * |
