# Supplementary material for: Locally adapted gut microbiomes mediate host stress tolerance
Source: ISME J. 2021 Mar 3;15(8):2401–14. doi: 10.1038/s41396-021-00940-y (PMC8319338; doi:10.1038/s41396-021-00940-y)
Supplement: Supplementary file 7 — Table SI7 [file 41396_2021_940_MOESM7_ESM.docx]

Table SI7

|  | *F* | df | df.res | *p*-value |
| --- | --- | --- | --- | --- |
| Time | 1114.5867 | 3 | 248 | <0.0001 *** |
| Diet | 1.4975 | 1 | 248 | 0.222223 |
| Microbiome type | 0.0449 | 1 | 656.67 | 0.832314 |
| Genotype | 5.9080 | 7 | 648.40 | <0.0001 *** |
| Time x Diet | 1.0481 | 3 | 248 | 0.371911 |
| Time x Microbiome type | 1.4794 | 3 | 248 | 0.220658 |
| Diet x Microbiome type | 6.5135 | 1 | 248 | 0.011306 * |
| Time x Genotype | 2.0407 | 21 | 248 | 0.005587 ** |
| Diet x Genotype | 1.1571 | 7 | 248 | 0.328219 |
| Microbiome type x Genotype | 1.9660 | 7 | 617.22 | 0.057460 |
| Time x Diet x Microbiome type | 1.1095 | 3 | 248 | 0.345847 |
| Time x Diet x Genotype | 0.7811 | 21 | 248 | 0.741880 |
| Time x Microbiome type x Genotype | 0.9332 | 21 | 248 | 0.548387 |
| Diet x Microbiome type x Genotype | 1.9344 | 7 | 248 | 0.064778 · |
| Time x Diet x Microbiome type x Genotype | 0.5047 | 21 | 248 | 0.967052 |
