# Supplementary material for: Locally adapted gut microbiomes mediate host stress tolerance
Source: ISME J. 2021 Mar 3;15(8):2401–14. doi: 10.1038/s41396-021-00940-y (PMC8319338; doi:10.1038/s41396-021-00940-y)
Supplement: Supplementary file 8 — Table SI8 [file 41396_2021_940_MOESM8_ESM.docx]

Table SI8

|  | Survival | Fecundity | Body size day 10 | Shannon entropy |
| --- | --- | --- | --- | --- |
| Survival |  |  |  |  |
| Fecundity | *r*=-0.05636138  *t*=-0.21122  df=14  *p*=0.8358 |  |  |  |
| Body size day 10 | *r*=-0.4872618  *t*=-2.0878  df=14  *p*=0.05557 | *r*=-0.05982255  *t*=-0.22424  df=14  *p*=0.8258 |  |  |
| Shannon entropy | *r*=0.1103409  *t*=0.41539  df=14  *p*=0.6841 | *r*=0.1909124  *t*=0.72771  df=14  *p*=0.4788 | *r*=-0.06545091  *t*=-0.24542  df=14  *p*=0.8097 |  |
