# Supplementary material for: Locally adapted gut microbiomes mediate host stress tolerance
Source: ISME J. 2021 Mar 3;15(8):2401–14. doi: 10.1038/s41396-021-00940-y (PMC8319338; doi:10.1038/s41396-021-00940-y)
Supplement: Supplementary file 9 — Table SI9 [file 41396_2021_940_MOESM9_ESM.docx]

Table SI9

| OTU | Class | Log2FoldChange | *P*adj |
| --- | --- | --- | --- |
| OTU1-Burkholderiaceae | Gammaproteobacteria | -2.858439 | <0.00001 *** |
| OTU5-Candidatus Hepatincola sp. | Alphaproteobacteria | 2.627916 | <0.00001 *** |
| OTU6-Flavobacterium sp. | Bacteroidia | -2.989165 | <0.00001 *** |
| OTU12-Staphylococcus sp. | Bacilli | 6.366721 | <0.00001 *** |
| OTU13-Candidatus Limnoluna sp. | Actinobacteria | 8.078177 | <0.00001 *** |
| OTU22-Luteolibacter sp. | Verrucomicrobiae | 4.702418 | <0.00001 *** |
| OTU47-Rubinisphaeraceae | Planctomycetacia | 8.663749 | <0.00001 *** |
