# Supplementary material for: Locally adapted gut microbiomes mediate host stress tolerance
Source: ISME J. 2021 Mar 3;15(8):2401–14. doi: 10.1038/s41396-021-00940-y (PMC8319338; doi:10.1038/s41396-021-00940-y)
Supplement: Supplementary file 10 — Table SI10 [file 41396_2021_940_MOESM10_ESM.docx]

Table SI10

|  | *F* | df | df.res | *p*-value |
| --- | --- | --- | --- | --- |
| Diet | 35.2519 | 1 | 4 | 0.0040347 ** |
| Microbiome type | 89.9831 | 2 | 9.37 | <0.0001*** |
| Genotype | 6.8175 | 8 | 423.24 | <0.0001*** |
| Microbiome type x Diet | 26.6568 | 2 | 4 | 0.0048709 ** |
| Diet x Genotype | 85.7493 | 7 | 4 | 0.0003413 *** |
| Microbiome type x genotype | 171.9533 | 6 | 4 | <0.0001*** |
| Diet x Microbiome type x Genotype | 131.9702 | 5 | 4 | 0.0001579 *** |
