# Supplementary material for: Locally adapted gut microbiomes mediate host stress tolerance
Source: ISME J. 2021 Mar 3;15(8):2401–14. doi: 10.1038/s41396-021-00940-y (PMC8319338; doi:10.1038/s41396-021-00940-y)
Supplement: Supplementary file 11 — Table SI11 [file 41396_2021_940_MOESM11_ESM.docx]

Table SI11

|  | *F* | *R²* | df | *p*-value |
| --- | --- | --- | --- | --- |
| Donor-Recipient | 11.0436 | 0.26177 | 1 | 0.001 *** |
| Microbiome type | 1.1603 | 0.02750 | 1 | 0.326 |
| Diet | 0.3502 | 0.00830 | 1 | 0.986 |
| Genotype | 1.0323 | 0.19575 | 8 | 0.448 |
| Donor-Recipient x Diet | 0.3894 | 0.00923 | 1 | 0.969 |
| Diet x Microbiome type | 0.9923 | 0.02352 | 1 | 0.434 |
| Diet x Genotype | 0.7231 | 0.11998 | 7 | 0.920 |
| Microbiome type x Genotype | 1.0430 | 0.14833 | 6 | 0.451 |
| Diet x Microbiome type x Genotype | 0.9350 | 0.11081 | 5 | 0.577 |
