# Supplementary material for: Locally adapted gut microbiomes mediate host stress tolerance
Source: ISME J. 2021 Mar 3;15(8):2401–14. doi: 10.1038/s41396-021-00940-y (PMC8319338; doi:10.1038/s41396-021-00940-y)
Supplement: Supplementary file 12 — Table SI12 [file 41396_2021_940_MOESM12_ESM.docx]

Table SI12

|  | *F* | df | *p*-value |
| --- | --- | --- | --- |
| Diet | 0.3083 | 1 | 0.580203 |
| Microbiome type | 2.8744 | 1 | 0.093705 |
| Genotype | 4.3683 | 2 | 0.015674 * |
| Microbiome type x Diet | 7.1474 | 1 | 0.009019 ** |
| Diet x Genotype | 1.5098 | 2 | 0.226884 |
| Microbiome type x genotype | 3.9019 | 2 | 0.023965 * |
| Diet x Microbiome type x Genotype | 7.3070 | 2 | 0.001187 ** |
