# Supplementary figures and images for: Locally adapted gut microbiomes mediate host stress tolerance
Source: ISME J. 2021 Mar 3;15(8):2401–14. doi: 10.1038/s41396-021-00940-y (PMC8319338; doi:10.1038/s41396-021-00940-y)

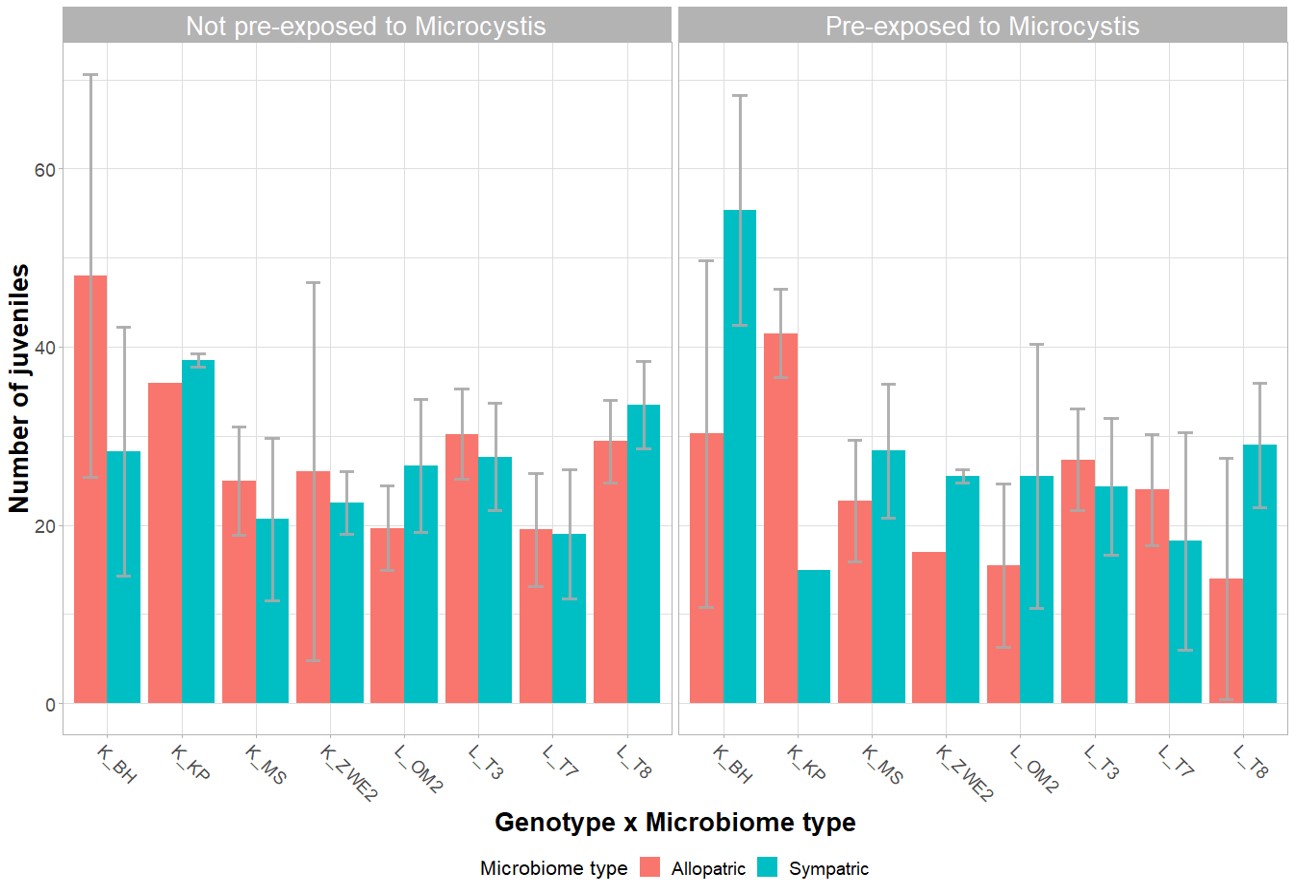

Supplement: Supplementary file 13 — Figure SI1 [file 41396_2021_940_MOESM13_ESM.jpg]

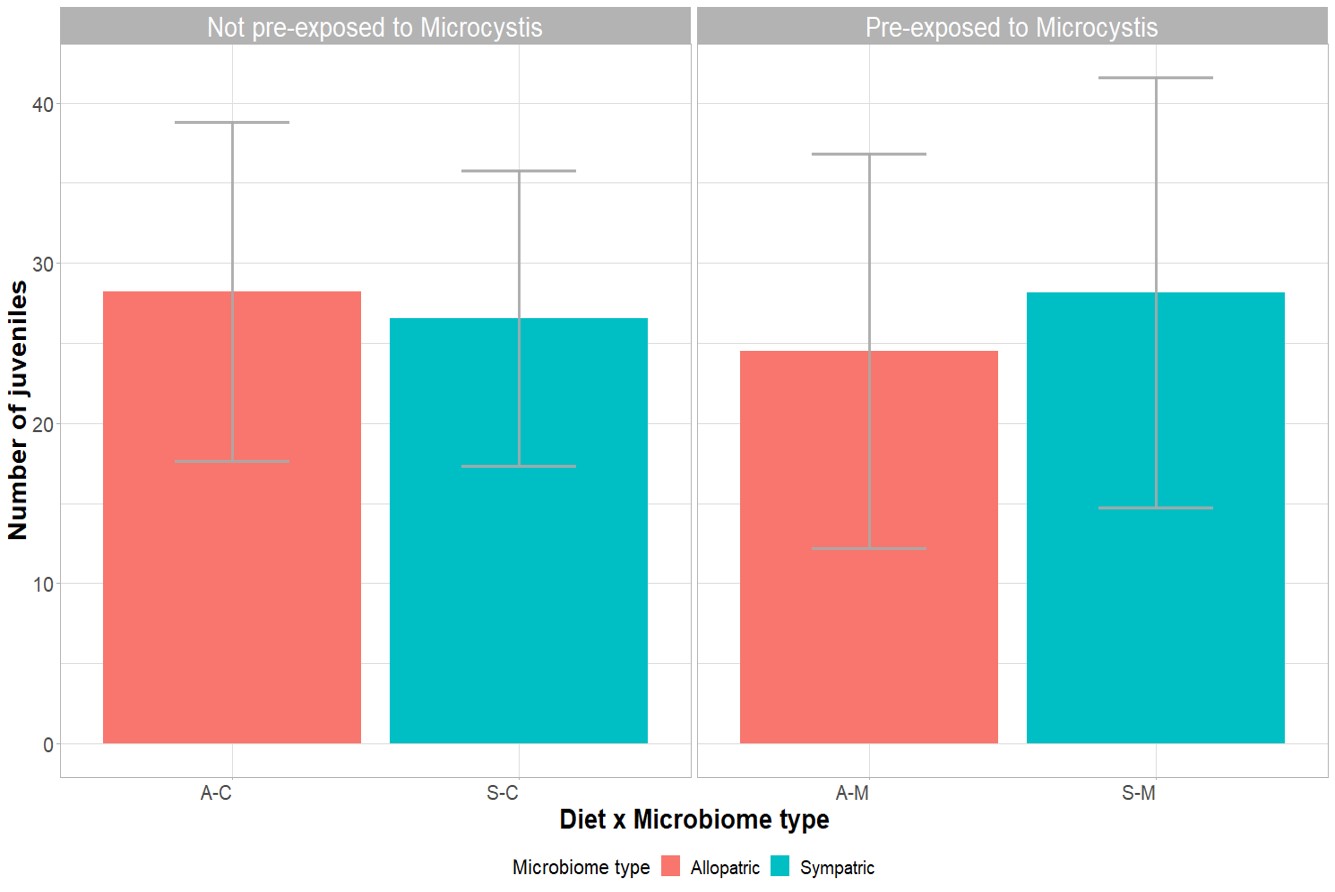

Supplement: Supplementary file 14 — Figure SI2 [file 41396_2021_940_MOESM14_ESM.jpg]

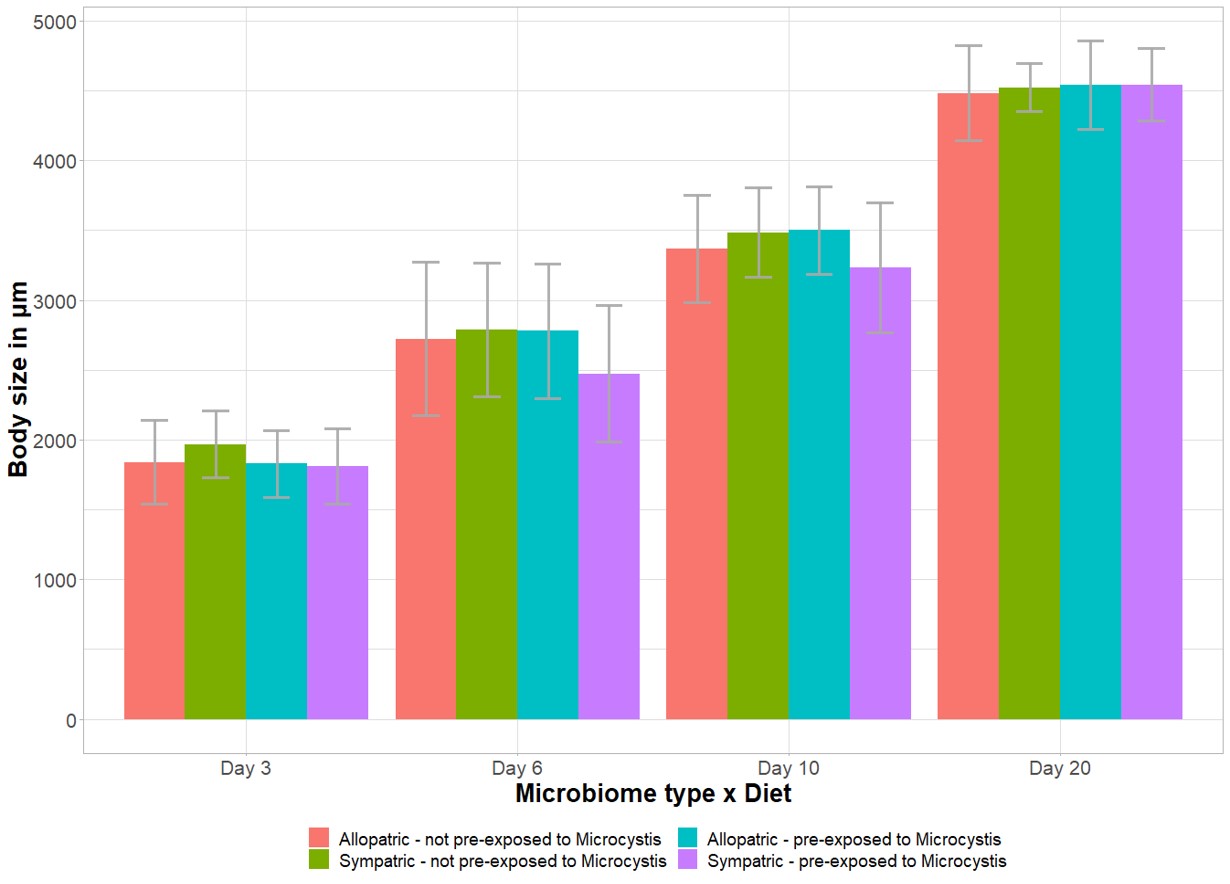

Supplement: Supplementary file 15 — Figure SI3 [file 41396_2021_940_MOESM15_ESM.jpg]

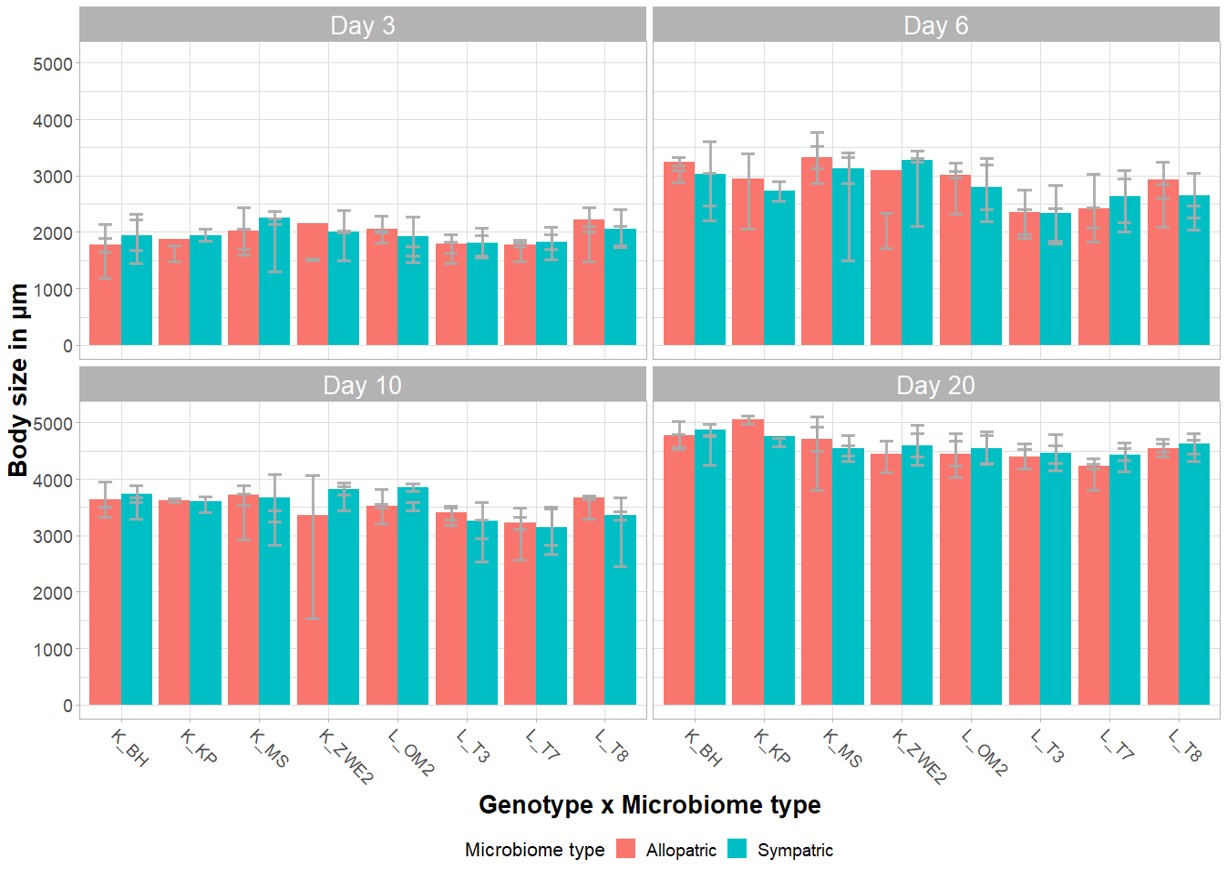

Supplement: Supplementary file 16 — Figure SI4 [file 41396_2021_940_MOESM16_ESM.jpg]

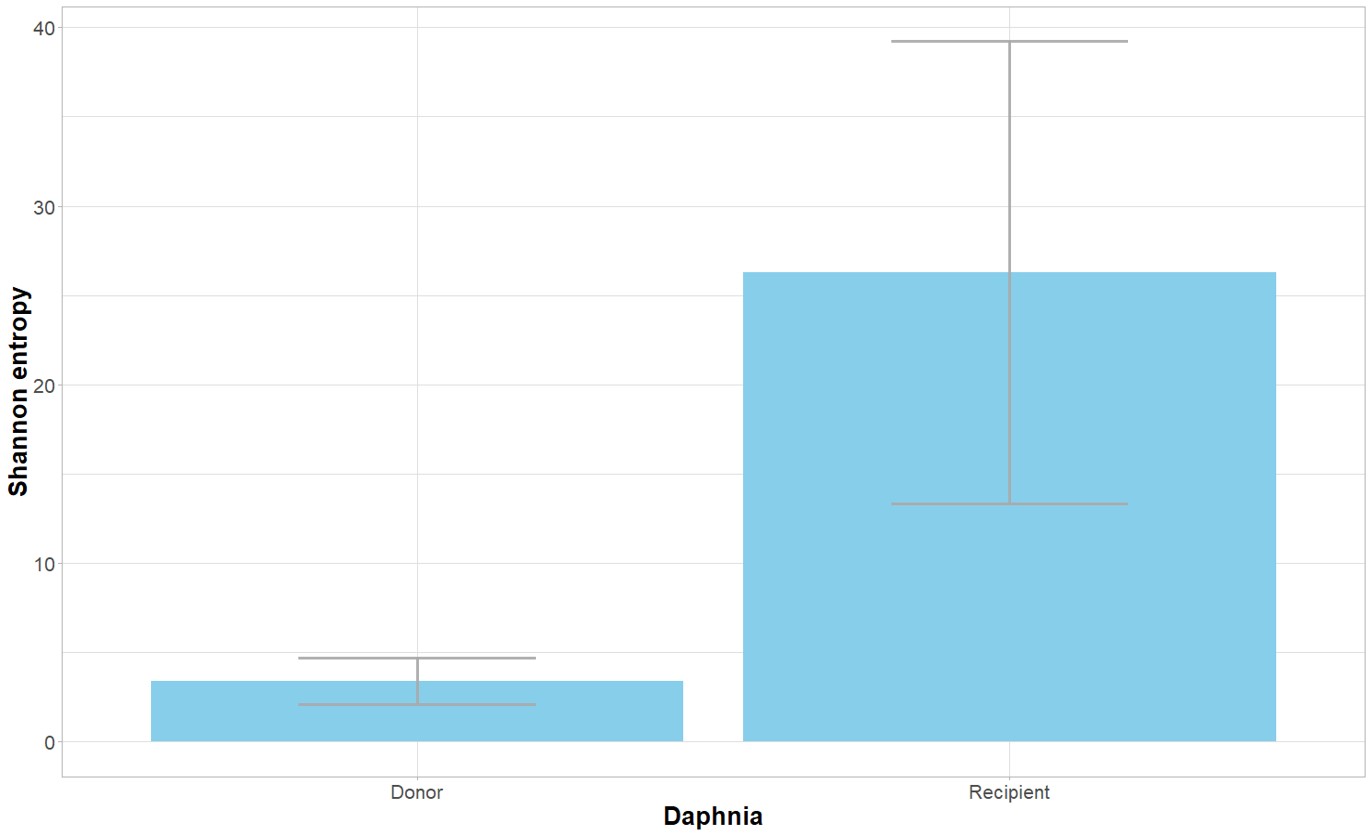

Supplement: Supplementary file 17 — Figure SI5 [file 41396_2021_940_MOESM17_ESM.jpg]

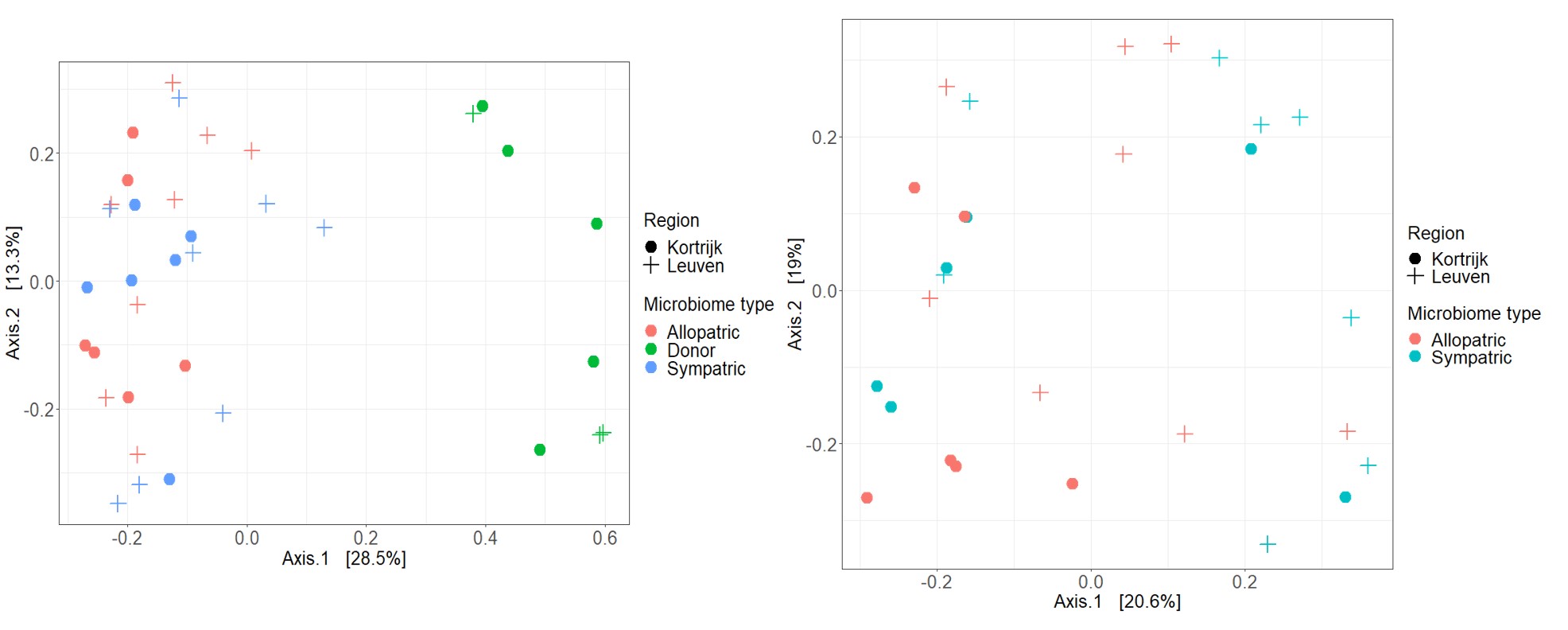

Supplement: Supplementary file 18 — figure SI6 [file 41396_2021_940_MOESM18_ESM.jpg]

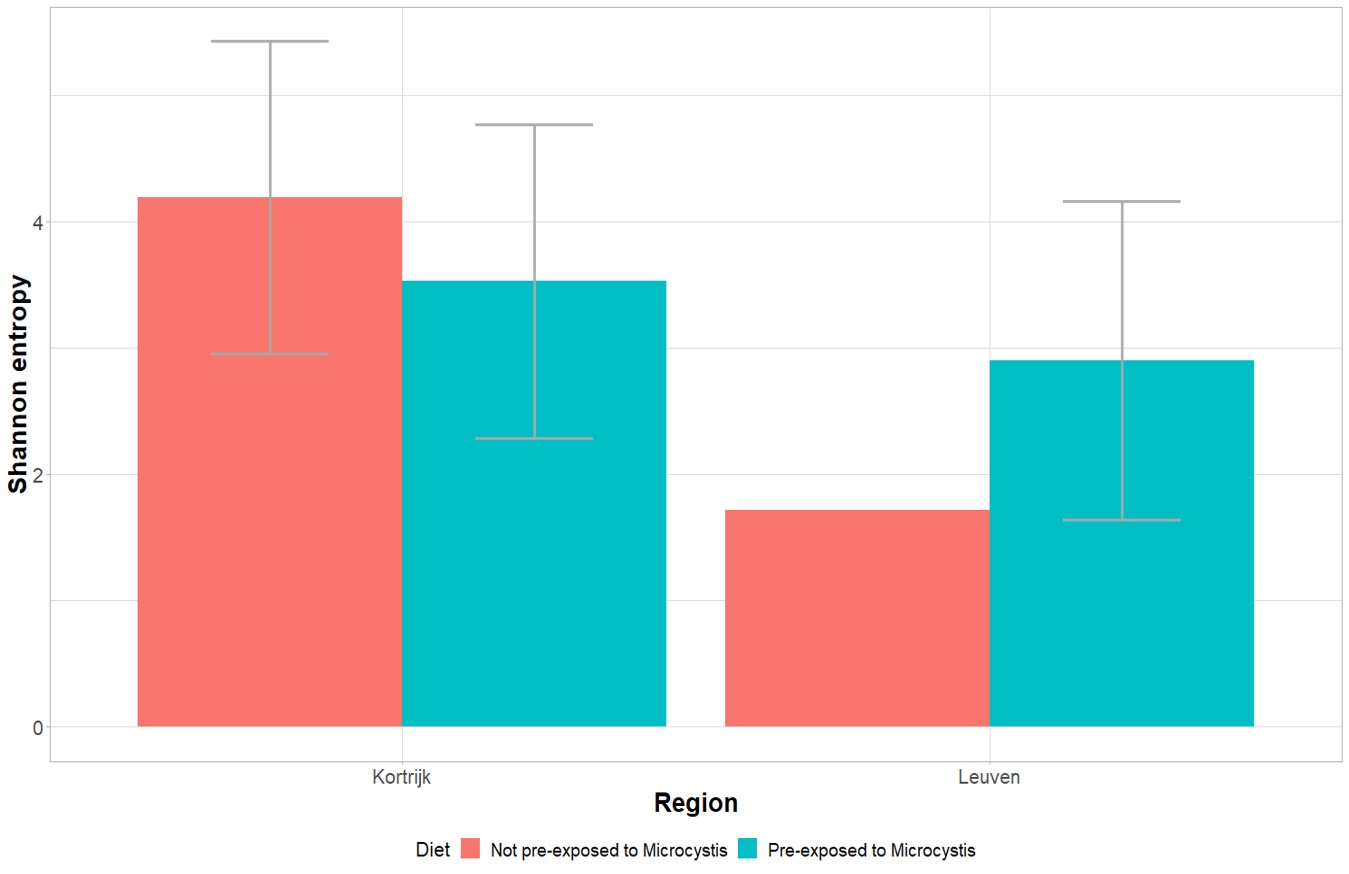

Supplement: Supplementary file 19 — Figure SI7 [file 41396_2021_940_MOESM19_ESM.jpg]

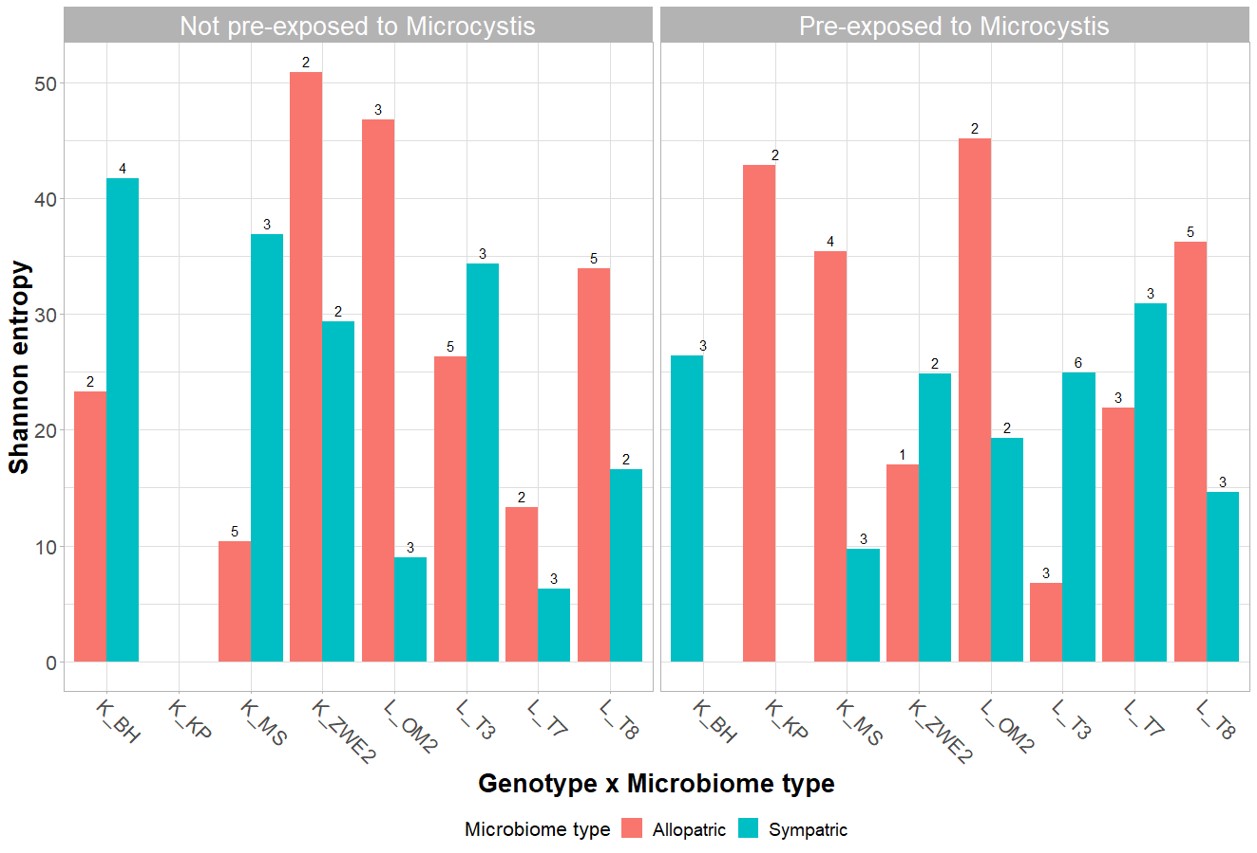

Supplement: Supplementary file 20 — Figure SI8 [file 41396_2021_940_MOESM20_ESM.jpg]
